# A Temporal PROTAC Cocktail‐Mediated Sequential Degradation of AURKA Abrogates Acute Myeloid Leukemia Stem Cells

**DOI:** 10.1002/advs.202104823

**Published:** 2022-06-02

**Authors:** Fang Liu, Xuan Wang, Jianli Duan, Zhijie Hou, Zhouming Wu, Lingling Liu, Hanqi Lei, Dan Huang, Yifei Ren, Yue Wang, Xinyan Li, Junxiao Zhuo, Zijian Zhang, Bin He, Min Yan, Huiming Yuan, Lihua Zhang, Jinsong Yan, Shijun Wen, Zifeng Wang, Quentin Liu

**Affiliations:** ^1^ Sun Yat‐sen University Cancer Center State Key Laboratory of Oncology in South China Collaborative Innovation Center for Cancer Medicine Guangzhou 510060 China; ^2^ Institute of Cancer Stem Cell Dalian Medical University Dalian 116044 China; ^3^ Department of Hematology the Third Affiliated Hospital of Sun Yat‐sen University Guangzhou 510630 China; ^4^ Department of Urology Kidney and Urology Center Pelvic Floor Disorders Center The Seventh Affiliated Hospital Sun Yat‐sen University Shenzhen 518000 China; ^5^ Department of Hematology the Second Affiliated Hospital of Dalian Medical University Dalian 116027 China; ^6^ CAS Key Laboratory of Separation Sciences for Analytical Chemistry National Chromatographic R&A Center Dalian Institute of Chemical Physics Chinese Academy of Sciences Dalian 116023 China

**Keywords:** acute myeloid leukemia stem cells, Aurora kinase A (AURKA), PROTAC cocktail, E3 ubiquitin ligase

## Abstract

AURKA is a potential kinase target in various malignancies. The kinase‐independent oncogenic functions partially disclose the inadequate efficacy of the kinase inhibitor in a Phase III clinical trial. Simultaneously targeting the catalytic and noncatalytic functions of AURKA may be a feasible approach. Here, a set of AURKA proteolysis targeting chimeras (PROTACs) are developed. The CRBN‐based dAurA383 preferentially degrades the highly abundant mitotic AURKA, while cIAP‐based dAurA450 degrades the lowly abundant interphase AURKA in acute myeloid leukemia (AML) cells. The proteomic and transcriptomic analyses indicate that dAurA383 triggers the “mitotic cell cycle” and “stem cell” processes, while dAurA450 inhibits the “MYC/E2F targets” and “stem cell” processes. dAurA383 and dAurA450 are combined as a PROTAC cocktail. The cocktail effectively degrades AURKA, relieves the hook effect, and synergistically inhibits AML stem cells. Furthermore, the PROTAC cocktail induces AML regression in a xenograft mouse model and primary patient blasts. These findings establish the PROTAC cocktail as a promising spatial‐temporal drug administration strategy to sequentially eliminate the multifaceted functions of oncoproteins, relieve the hook effect, and prevent cancer stem cell‐mediated drug resistance.

## Graphical abstract

This study establishes the PROTAC cocktail of CRBN‐based dAurA383 and cIAP‐based dAurA450 as a promising spatial‐temporal drug administration strategy to sequentially eliminate the multifaceted functions of mitotic AURKA and interphase AURKA, relieve the hook effect, and prevent cancer stem cell‐mediated drug resistance in acute myeloid leukemia. PROTAC cocktail strategy is proposed to be a second generation of PROTACs.

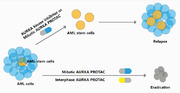

## 1 Introduction

Aurora kinase A (AURKA, Aurora‐A) is mainly localized in the spindle poles and plays a crucial role in proper bipolar spindle formation in the mitotic process.^[^ ^1^, ^2^ ^]^ AURKA is frequently overexpressed in various malignancies, including acute myeloid leukemia (AML).^[^ ^3^, ^4^, ^5^ ^]^ A high level of AURKA is associated with a higher tumor grade and poorer prognosis.^[^ ^6^, ^7^, ^8^ ^]^ Recently, we and others have demonstrated that AURKA is highly expressed in CD34^+^/CD38^−^ AML stem cells and augments stemness.^[^ ^9^, ^10^, ^11^, ^12^ ^]^ The genetic depletion of AURKA inhibits proliferation, impairs self‐renewal and induces apoptosis of AML stem cells.^[^ ^12^ ^]^ Therefore, AURKA is considered a potential therapeutic cancer target in AML. We previously developed a set of AURKA kinase inhibitors with anticancer effects in various cancers, including AML.^[^ ^13^, ^14^, ^15^, ^16^, ^17^, ^18^, ^19^ ^]^ A series of AURKA kinase inhibitors, including alisertib (MLN8237), danusertib, and ENMD‐2076, have also been evaluated in preclinical and clinical studies.^[^ ^8^ ^]^ However, the completed phase II/III clinical trials have been regarded as failures due to their limited efficacy over existing drugs.^[^ ^20^, ^21^ ^]^

Recent studies indicate that AURKA kinase inhibitors maybe unable to abrogate kinase‐independent oncogenic functions. We found that the nuclear translocation of AURKA in the interphase facilitates stem‐cell‐like phenotypes via directly activating c‐Myc^[^ ^22^ ^]^ and FOXM1 transcription,^[^ ^23^ ^]^ or by enhancing N^6^‐methyladenosine modification of DROSHA mRNA to activate STC1 transcription^[^ ^24^ ^]^ in a kinase‐independent manner. Other groups have found that AURKA directly binds to c‐Myc^[^ ^25^ ^]^ and N‐myc^[^ ^26^, ^27^ ^]^ proto‐oncoproteins thereby noncatalytically protecting them from proteasomal degradation. Thus, a strategy of simultaneously targeting the kinase‐dependent and kinase‐independent functions of AURKA to induce mitotic catastrophe and impair cancer stemness is proposed to be a more effective therapeutic approach.

The emerging proteolysis targeting chimeras (PROTACs) provide an opportunity to target both the catalytic and noncatalytic functions of kinases via artificial ubiquitylation and subsequent degradation by proteasomes.^[^ ^28^ ^]^ The bifunctional small molecule PROTACs are comprised of two chemical moieties. One binds to a target of interest and the other to a cellular E3‐ubiquitin ligase. Mitotic AURKA naturally undergoes ubiquitination by anaphase‐promoting complex/cyclosome (APC/C) ubiquitin ligase with its coactivator CDH1,^[^ ^29^ ^]^ or Skp1‐Cul1‐F‐box proteinubiquitin ligase with its coactivator FBXL7/FBXW7^[^ ^26^, ^30^, ^31^ ^]^ and is subsequently degraded upon mitotic exit. The von Hippel‐Lindau (VHL) is reported to ubiquitinate AURKA for degradation in quiescent cells.^[^ ^32^ ^]^ However, the E3 ligase which could intrinsically recognize and degrade interphase AURKA has not been uncovered. Recent studies have reported CRBN‐based PROTACs could effectively degrade AURKA, cause an S‐phase defect^[^ ^33^ ^]^ and prevent fragmentation of the mitochondrial network.^[^ ^34^ ^]^ Yet, these AURKA PROTACs confront the problem of the hook effect, and no evidence indicates that AURKA PROTACs could eliminate the stemness promoting function of interphase AURKA.

Here, we have developed a set of AURKA PROTACs based on the E3 ligases CRBN, cIAP, and VHL. The CRBN‐based PROTAC dAurA383 preferentially degrades the highly abundant mitotic AURKA, while cIAP‐based PROTAC dAurA450 degrades the lowly abundant interphase AURKA. The combination of dAurA383 and dAurA450 sequentially degrades the mitotic and interphase AURKA, impairs AML proliferation, relieves the hook effect, and abrogates acute myeloid leukemia stem cells. This study extends the spatial‐temporal drug administration strategy of PROTACs.

## 2 Results

### 2.1 Design and Characterization of Different E3 Ubiquitin Ligase‐Based AURKA PROTACs

**Figure 1:**
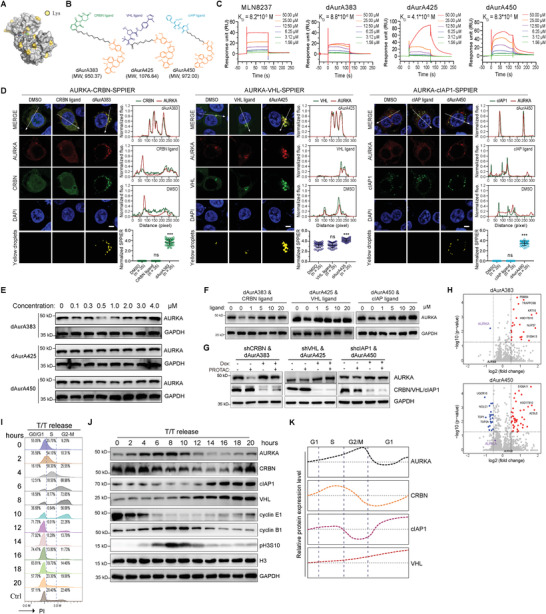
Design and characterization of different E3 ubiquitin ligase‐based AURKA PROTACs. A) Crystal structure of AURKA in complex with MLN8054 (PDB code 2×81). The lysine residues on the surface of AURKA are highlighted. B) Chemical structures of AURKA PROTAC based on MLN8237 and the ligands of CRBN, VHL, and cIAP. MW, molecular weight (Daltons). C) SPR sensorgrams and KD values of MLN8237 and AURKA PROTACs binding to the AURKA protein. D) Dual‐colored fluorescence SPPIER (DF‐SPPIER) imaging of ternary complex formation in HEK293T cells with PROTACs and E3 ligands induction (500 × 10^−9^ m) for 3 h. Fluorescence histogram of the line across the cells (normalized fluo., normalized fluorescence intensity) and the log10 normalized sum of pixel fluorescence intensity (normalized SPPIER, log_10_ (intensity + 1)) of yellow droplets in each cell are shown on the right. Scale bar: 5 µm. E) Degradation of endogenous AURKA in KG1A cells following 6 h treatment with the indicated concentration of PROTACs. F) Degradation of endogenous AURKA in KG1A cells following 6 h treatment with the indicated PROTACs (500 × 10^−9^ m) and E3 ligase ligands. G) Degradation of endogenous AURKA in KG1A cells with or without doxycycline (DOX, 0.5 µg mL^−1^) treatment for 72 h following 6 h treatment with the indicated PROTACs (500 × 10^−9^ m). H) TMT‐based quantitative proteomics after treatment with dAurA383 (500 × 10^−9^ m), dAurA450 (500 × 10^−9^ m) or the DMSO Vehicle for 6 h in KG1A cells. The differentially expressed proteins are presented in the volcano plot. I. KG1A cells were synchronized at the G1/S boundary by a double thymidine method, released into fresh media, and harvested at the indicated times (T/T release). The cell cycle profile was assayed by FACS with propidium iodide (PI) staining. Ctrl, proliferating KG1A cells under normal cell culture condition were used as control. J) The protein expression levels of the indicated proteins after T/T release were measured by Western blot. K) Schematic depiction of the relative protein levels of AURKA, CRBN, cIAP1, and VHL in KG1A cells. Statistics, significance: one‐way ANOVA with Bonferroni correction (D); ns, not significant; ***P < 0.001.

We checked the crystal structures of AURKA in the Protein Data Bank^[^ ^35^ ^]^ to evaluate its degradation potential and to find its binding scaffold (**Figure** **1**A, PDB code 2×81). Considering that lysine residues on protein surface are accessible for modification,^[^ ^36^, ^37^ ^]^ the abundant surface lysine residues suggest AURKA is a potential candidate for PROTAC mediated ubiquitination degradation. MLN8054 has been reported to selectively bind to the pocket of AURKA.^[^ ^38^, ^39^ ^]^ MLN8237 is a close analogue of MLN8054 with greater tolerable safety profile in multiple clinical studies^[^ ^40^ ^]^ (Figure S1A, Supporting Information). Considering the greater binding affinity of MLN8237,^[^ ^41^ ^]^ we chose MLN8237 as the AURKA binding scaffold. The commonly used CRBN ligand, VHL ligand, and cIAP ligand were attached to MLN8237 through different linkers to synthesize a series of AURKA PROTACs (Figure 1B; Figure S1B, Supporting Information). The structure and purity of the compounds are shown in the Experimental Section. We set out to investigate the degradation activity of these PROTACs against AURKA by Western blot. As shown in Figure S1C,D of the Supporting Information, CRBN‐based dAurA383 and dAurA1071 exhibit the best degradation activity in all of the PROTACs. Besides, the VHL‐based dAurA425 and the cIAP‐based dAurA450 exhibit moderate degradation activity. We chose dAurA383, dAurA425, and dAurA450 with distinct E3 ligase as the potential candidates for further evaluation.

The surface plasmon resonance (SPR) analysis demonstrates that all of the three PROTACs display comparable binding capacity to AURKA proteins compared with MLN8237 (Figure 1C and Figure S1E (Supporting Information), MLN8237 K_D_ = 8.2*10^−5^ m, dAurA383 K_D_ = 8.8*10^−6^ m, dAurA425 K_D_ = 4.1*10^−5^ m, dAurA450 K_D_ = 8.3*10^−5^ m). In addition, we detected the ternary complexes formation of [AURKA‐PROTACs‐E3 ubiquitin ligases] in HEK293T cells using separation of phases‐based protein interaction reporter (SPPIER) assays.^[^ ^42^, ^43^ ^]^ Considering the strong background foci of AURKA and cIAP1 caused by autoaggregation (Figure S1F, Supporting Information), we optimized the EGFP‐based SPPIER assays to dual‐colored fluorescence SPPIER (DF‐SPPIER) using mCherry to label AURKA and EGFP to label E3 ubiquitin ligase to examine the ternary complexes formation of [AURKA‐dAurA383‐CRBN], [AURKA‐dAurA425‐VHL], and [AURKA‐dAurA450‐cIAP1]. dAurA383, dAurA425, and dAurA450 induce the formation of highly coincident peaks of AURKA and E3 ubiquitin ligase and stronger yellow fluorescent droplets (Figure 1D), indicating ternary complexes formation. Notably, the yellow foci are observed in the control AURKA‐VHL‐SPPIER cells with dimethyl sulfoxide (DMSO) or VHL ligands treatment (Figure 1D), while no foci are detected in VHL‐EGFP‐HOTag6 cells (Figure S1F, Supporting Information). The intrinsic interaction of VHL and AURKA^[^ ^32^ ^]^ might lead to the SPPIER phase formation in the control AURKA‐VHL‐SPPIER cells.

We treated KG1A cells with various concentrations of these compounds. Western blot was used to quantify the protein levels of residual AURKA upon treatment of PROTACs. The optimal degradation concentrations of dAurA383, dAurA425, and dAurA450 are 500 × 10^−9^, 1000 × 10^−9^, and 1000 × 10^−9^ m in KG1A cells, respectively (Figure 1E). All three PROTACs display the hook effect. The degradation activity of dAurA383, dAurA425, and dAurA450 was also confirmed in Kasumi‐1, HL60, and U937 cells (Figure S1G, Supporting Information). The time‐course studies demonstrate that dAurA383, dAurA425, and dAurA450 achieve optimal degradation within 6 h (Figure S1H, Supporting Information). Supplement of the E3 ligands and knockdown of E3 ligases restore AURKA expression degraded by PROTACs, respectively (Figure 1F,G). Furthermore, the degradation activity of these PROTACs is blocked by MLN4924 (an NEDD8‐activating enzyme inhibitor), Bortezomib, and MG132 (proteasome inhibitors) (Figure S1I,J, Supporting Information).

To further examine the selective degradation effect of the three PROTACs, we performed tandem mass tags (TMTs)‐based quantitative proteomic analysis after treatment with dAurA383, dAurA425, dAurA450 or DMSO vehicle in KG1A cells. dAurA383 displays a highly specific degradation activity on AURKA, while dAurA425 and dAurA450 are less specific (Figure 1H; Figure S1K and Table S1, Supporting Information).

Although the results of the SPR and SPPIER assays demonstrate that all of dAurA383, dAurA425, and dAurA450 bind AURKA and form ternary complexes with E3 ligases, they have significantly different degradation efficiencies. We speculated that the cell cycle‐dependent expression of AURKA and E3 ligases might contribute to this discrepancy and examined the cell cycle phase distribution of AURKA, CRBN, VHL, and cIAP1 in KG1A and Kasumi‐1 cells (Figure 1I,J; Figure S2A,B, Supporting Information). The protein expression patterns of AURKA, CRBN, cIAP1, and VHL are summarized in Figure 1K and Figure S2C (Supporting Information). The expression of CRBN increases when cells enter the S phase and reaches a maximum level at the S/G2 boundary (KG1A cells) or G2/M boundary (Kasumi‐1 cells), then reduces along with mitosis progression and increases again when cells enter the next cell cycle. This observation suggests CRBN‐based dAurA383 could timely and effectively degrade the newly synthesized AURKA proteins from the S phase to mitosis. The expression of cIAP1 and VHL increases when cells exit mitosis and enter the G1 phase. The expression patterns of the CRBN, cIAP1, and VHL in the cell cycle support the CRBN‐based PROTAC dAurA383 effectively degrades the highly abundant mitotic AURKA, while the cIAP‐based PROTAC dAurA450 degrades the lowly abundant interphase AURKA.

### 2.2 AURKA PROTACs Inhibit Cell Growth and Induce Apoptosis of AML Cells In Vitro

**Figure 2:**
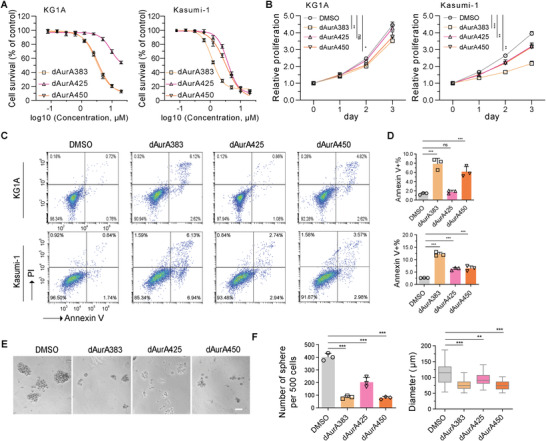
AURKA PROTACs inhibit cell growth and induce apoptosis of AML cells in vitro. A) Dose–response curves of KG1A or Kasumi‐1 treated with AURKA PROTACs. Cells were treated with various concentrations of AURKA PROTACs for 72 h and were stained with CCK8. For IC50 in KG1A cells, dAurA383 = 3.04 ± 0.28 × 10^−6^ m, dAurA425 = 9.23 ± 2.00 × 10^−6^ m, dAurA450 = 3.41 ± 0.28 × 10^−6^ m. For IC50 in Kasumi‐1 cells, dAurA383 = 1.17 ± 0.08 × 10^−6^ m, dAurA425 = 3.75 ± 0.32 × 10^−6^ m, dAurA450 = 3.19 ± 0.35 × 10^−6^ m. B) Cell proliferation of KG1A or Kasumi‐1 treated with AURKA PROTACs. Cells were treated with AURKA PROTACs (1 × 10^−6^ m) and were stained with CCK8. C,D) Cell apoptosis of KG1A cells treated with AURKA PROTACs (1 × 10^−6^ m) for 48 h. E,F) Methylcellulose‐based colony forming cell (CFC) assays show the effects of AURKA PROTACs (1 × 10^−6^ m) on KG1A cells for 14 days. Sphere number (diameter > 50 µm, left panel) and size (diameter, right panel) were calculated. Scale bar: 50 µm. Statistics, significance: one‐way ANOVA with Bonferroni correction (B,D,F); ns, not significant; *P < 0.05; **P < 0.01; ***P < 0.001.

To study the anticancer activity of these PROTACs in vitro, KG1A, Kasumi‐1, HL60, NB4, U937, and THP1 cells were treated with dAurA383, dAurA425, and dAurA450. The inhibition of cell proliferation was evaluated by CCK8 assays. In KG1A cells, the IC_50_ doses are 3.04 ± 0.28 × 10^−6^ m (dAurA383), 9.23 ± 2.00 × 10^−6^ m (dAurA425) and 3.41 ± 0.28 × 10^−6^ m (dAurA450). In Kasumi‐1 cells, the IC_50_ doses are 1.17 ± 0.08 × 10^−6^ m (dAurA383), 3.75 ± 0.32 × 10^−6^ m (dAurA425), and 3.19 ± 0.35 × 10^−6^ m (dAurA450) (**Figure** **2**A,B). In other AML cells, the IC_50_ dose‐levels are between 0.42 × 10^−6^ and 1.70 × 10^−6^ m for dAurA383, between 7.28 × 10^−6^ and 11.99 × 10^−6^ m for dAurA425, and between 1.95 × 10^−6^ and 2.77 × 10^−6^ m for dAurA450 (Figure S3, Supporting Information). The apoptotic induction function of the three PROTACs was further assessed by Annexin V assays. dAurA383 and dAurA450 induce apoptosis of both KG1A and Kasumi‐1 cells, while dAurA425 weakly induces apoptosis of Kasumi‐1 cells (Figure 2C,D). In addition, methylcellulose‐based colony‐forming cell (CFC) assays demonstrate that the PROTACs significantly suppress the colony formation of KG1A cells (Figure 2E,F).

### 2.3 Characterization of Cellular Responses to PROTACs in AML Cells

**Figure 3:**
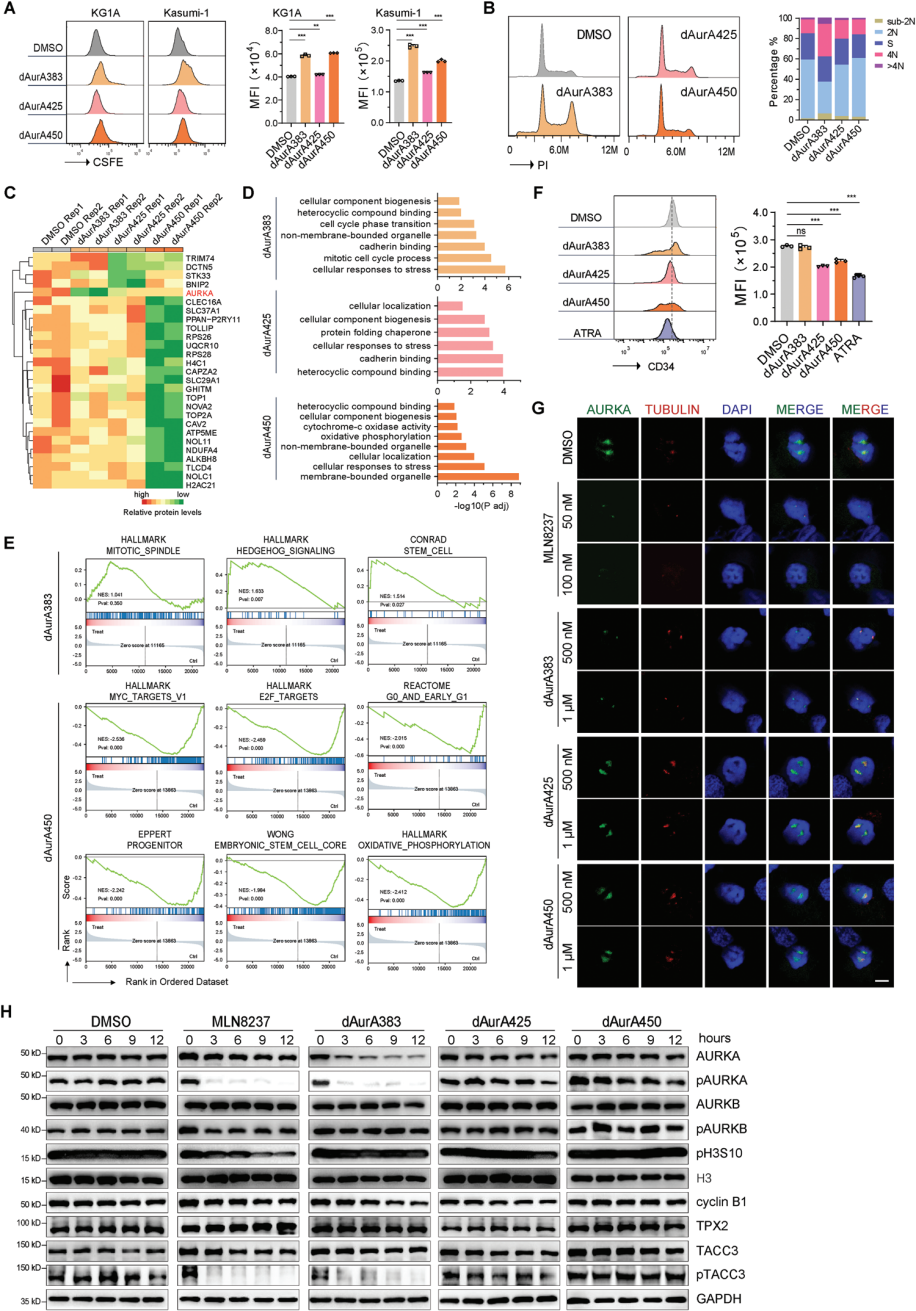
Characterization of cellular response to PROTACs in AML cells. A) KG1A and Kasumi‐1 cells were stained with a carboxyfluorescein succinimidyl amino ester (CFSE) probe and cultured with AURKA PROTACs (1 × 10^−6^ m) for 72 h. CFSE fluorescence was analyzed by flow cytometry. MFI, mean fluorescence intensity. B) KG1A cells were treated with AURKA PROTACs (1 × 10^−6^ m) for 48 h. The cell cycle profile was assayed by FACS with propidium iodide (PI) staining. The cell cycle phase distribution was analyzed by FlowJo software. C) A heatmap of the relative normalized abundance of proteins in TMT‐based quantitative proteomic assays. D) TMT‐based quantitative proteomics after treatment with PROTACs (500 × 10^−9^ m) or the DMSO Vehicle for 6 h in KG1A cells. The top 100 decreased proteins were subjected to g:Profiler to perform gene ontology (GO) analysis. E) Gene set enrichment analysis (GSEA) of RNA‐seq data from KG1A cells treated with AURKA PROTACs (1 × 10^−6^ m) for 48 h. F) KG1A cells were treated with AURKA PROTACs (1 × 10^−6^ m) or ATRA (0.6 × 10^−6^ m) for 48 h. Cell surface CD34 expression was analyzed by FACS. MFI, mean fluorescence intensity. G) KG1A cells were arrested with nocodazole (0.1 µg mL^−1^) for 16 h followed by PROTACs or MLN8237 treatment with indicated concentration for 6 h. AURKA localization were detected by immunofluorescence. Scale bar: 5 µm. H) KG1A cells were arrested with nocodazole (0.1 µg mL^−1^) for 16 h followed by DMSO, PROTACs (1 × 10^−6^ m) or MLN8237 (50 × 10^−9^ m) treatment for indicated times. The expression of AURKA and related proteins were detected. Statistics, significance: one‐way ANOVA with Bonferroni correction (A,F); ns, not significant; *P < 0.05; **P < 0.01; ***P < 0.001.

To explore the anticancer mechanism of the three PROTACs, we examined the cell cycle changes upon the PROATC treatment. The carboxyfluorescein succinimidyl amino ester (CFSE) staining assays demonstrate that dAurA383 and dAurA450 obviously delay the division of KG1A and Kasumi‐1 cells (**Figure** **3**A). The propidium iodide (PI) staining assays show that dAurA383 arrests the cell cycle in mitosis in KG1A and Kasumi‐1 cells, while dAurA450 does not (Figure 3B; Figure S4A, Supporting Information). Additionally, neither knockdown of E3 ligases nor treatment with E3 ligands changes the cell cycle phase distribution (Figure S4B,C, Supporting Information), suggesting the bifunctional PROTACs change the cell cycle through AURKA.

We profiled the transitory response of proteomics underlying dAurA383, dAurA425, and dAurA450 treatment (Table S1, Supporting Information). The differentially expressed proteins in each group (*p* < 0.05, abundance ratio < 0.5) are presented in a heatmap (Figure 3C). The top 100 decreased proteins in each group were subject to the g:Profiler^[^ ^44^ ^]^ for gene ontology (GO) analysis. The processes of “heterocyclic compound,” “cellular responses to stress,” and “cellular component biogenesis” are enriched in all the groups. Consistent with the cell cycle analysis, the processes of “cell cycle phase transition” and “mitotic cell cycle” are enriched in dAurA383 treated cells. The process of “oxidative phosphorylation” is enriched in dAurA450 treated cells (Figure 3D), supporting the emerging functions of interphase AURKA in mitochondrial dynamics and energy production.^[^ ^45^, ^46^ ^]^

We next evaluated the transcriptional responses of PROTAC treatment. The transcripts per million for each gene are listed in Table S2 of the Supporting Information. Principal component analysis of the top 1000 differentially expressed genes indicates a reasonable distribution and correlation for each sample (Figure S4D, Supporting Information). Gene set enrichment analysis (GSEA) shows dAurA383 treatment triggers “mitotic spindle” and “stem cell” processes, while dAurA450 treatment inhibits “MYC/E2F targets,” “stem cell,” and “oxidative phosphorylation” processes (Figure 3E). The transcriptomic profiling supports the finding that dAurA383 degrades mitotic AURKA, while dAurA450 degrades interphase AURKA. Consistent with changes of stemness in proteomics and transcriptomics, a population of KG1A cells increase cell surface CD34 (hematopoietic stem/progenitor cell marker^[^ ^47^ ^]^) in response to dAurA383 treatment, while most of the cells decrease cell surface CD34 in response to the dAurA450 treatment (Figure 3F).

We further investigated the effect of PROTACs on AURKA‐related signaling pathways. Consistent with the previous study of CRBN‐based PROTAC‐D,^[^ ^34^ ^]^ dAurA383 induces a marked degradation of the spindle associated AURKA, while preserves the centrosome associated AURKA (Figure 3G). Meanwhile, MLN8237 disrupts the localization of AURKA at the centrosome and spindle (Figure 3G). Furthermore, AURKB and cyclin B1 slightly decrease in mitotic arrest cells after dAurA383 treatment, suggesting a mitotic slippage in dAurA383 treated cells (Figure 3H). Although the expression of AURKA binding partners TPX2 and TACC3 are not affected, phosphorylated TACC3 (pTACC3 on S558) is significantly decreased in dAurA383 and MLN8237 treated cells (Figure 3H), implying that AURKA inhibition destroys the stabilization of parallel spindle microtubules.^[^ ^48^ ^]^

### 2.4 A dAurA383 and dAurA450 Cocktail Synergistically Inhibit the Growth and Stemness of AML Cells

The results from the CD34 flow cytometry assays and the global changes in proteomics and transcriptomics indicate that dAurA383 degrades the mitotic AURKA but triggers the stemness program activation in some cells, and that dAurA450 degrades interphase AURKA and confronts the stemness program. Therefore, we propose a PROTAC cocktail of dAurA383 and dAurA450 to synergistically induce AML regression by eliminating the proliferative cells and abrogating leukemia stem cells. The ratios of the PROTACs in cocktail were assessed. As shown in Figure S5A of the Supporting Information, the cocktail of dAurA383 and dAurA450 exerts the best cytotoxic effect at the ratio of either 1:1 or 0.75:1.25 in KG1A cells, while exhibits the best cytotoxic effect at the ratio of either 1.25:0.75, 1:1 or 0.75:1.25 in Kasumi‐1 cells. As these optimal ratios are close to 1:1, we used 1:1 to conduct all the PROTAC cocktail studies.

**Figure 4:**
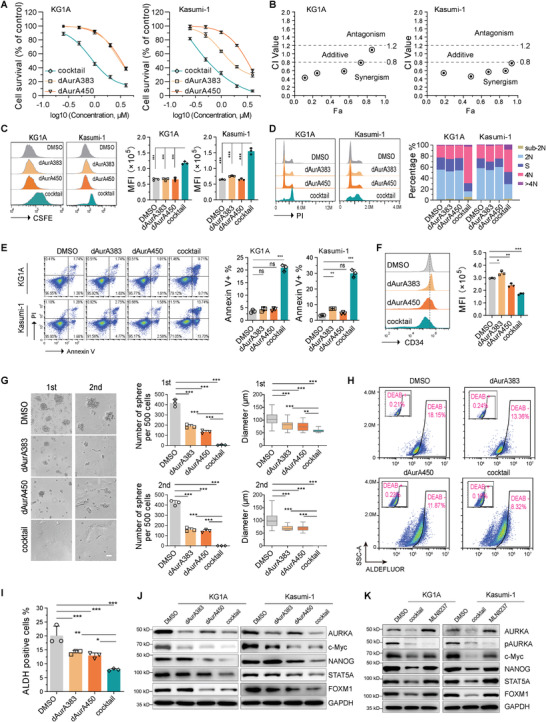
dAurA383 and dAurA450 synergistically inhibit the growth and stemness of AML cells. A,B) KG1A and Kasumi‐1 cells were treated with various concentrations of dAurA383, dAurA450 alone or in combination for 72 h. The growth inhibitory effects were determined by CCK8 staining. The CI value was calculated with CompuSyn software. C) KG1A and Kasumi‐1 cells were stained with carboxyfluorescein succinimidyl amino ester (CFSE) probe and cultured with PROTACs (500 × 10^−9^ m) for 72 h. CFSE fluorescence was analyzed by flow cytometry. MFI, mean fluorescence intensity. D) KG1A and Kasumi‐1 cells were treated with AURKA PROTACs (500 × 10^−9^ m) for 48 h. The cell cycle profile was assayed by FACS with propidium iodide (PI) staining. The cell cycle phase distribution was analyzed by FlowJo software. E) The apoptosis of KG1A and Kasumi‐1 cells treated with the indicated PROTACs (500 × 10^−9^ m) for 48 h. The rate of Annexin V positive cells was calculated. F) KG1A cells were treated with AURKA PROTACs (500 × 10^−9^ m) for 48 h. Cell surface CD34 expression was analyzed by FACS. MFI, mean fluorescence intensity. G) Methylcellulose‐based colony forming cell (CFC) assays to examine the stemness of KG1A cells treated with PROTACs (500 × 10^−9^ m) for 14 days. Sphere number (diameter > 50 µm, left panel) and size (diameter, right panel) were calculated. Scale bar: 50 µm. H,I) KG1A cells were treated with AURKA PROTACs (500 × 10^−9^ m) for 48 h. ALDH positive cells were analyzed by flow cytometry assays. J&K. KG1A and Kasumi‐1 cells were treated with AURKA PROTACs (1 × 10^−6^ m) and MLN8237 (50 × 10^−9^ m) for 48 h. Western blot analysis of AURKA as well as stemness markers c‐Myc, NANOG, STAT5A, and FOXM1. Statistics, significance: one‐way ANOVA with Bonferroni correction (A,C,E,F,G,I); ns, not significant; *P < 0.05; **P < 0.01; ***P < 0.001.

We investigated the cytotoxic effect of single and cocktail of PROTACs by CCK8 assays. PROTAC cocktail displays better antiproliferative action than that of the single drugs (**Figure** **4**A). These data were subjected to CompuSyn software to calculate the combination index (CI).^[^ ^49^ ^]^ An index less than 0.8, between 0.8 and 1.2, and more than 1.2 represents synergy, additivity, and antagonism, respectively.^[^ ^50^ ^]^ As shown in Figure 4B, dAurA383 and dAurA450 act synergistically to inhibit KG1A and Kasumi‐1 cells. The cocktail of dAurA383 and dAurA450 severely inhibits cell division (Figure 4C), arrests AML cells in mitosis and generates aneuploid cancer cells (Figure 4D). Annexin V assays further demonstrate that the PROTAC cocktail significantly triggers the apoptosis of KG1A and Kasumi‐1 cells compared with the single agent administration (Figure 4E).

The PROTAC cocktail effectively decreases cell surface CD34 (Figure 4F), suppresses both primary and secondary colony formation (Figure 4G) and reduces the ratio of aldehyde dehydrogenase (ALDH) positive AML stem cells (Figure 4H,I). The stemness markers c‐Myc, NANOG, STAT5A, and FOXM1 are decreased in the PROTAC cocktail treated AML cells (Figure 4J). Consistent with our finding that AURKA transcriptionally activate c‐Myc,^[^ ^22^ ^]^ the cocktail decreases c‐Myc at transcriptional level in KG1A and Kasumi‐1 cells (Figure S5B, Supporting Information). The cocktail also transcriptionally decreases MYCN and CD34 (Figure S5B, Supporting Information). Additionally, we compared the activity of MLN8237 and PROTAC cocktail in stemness inhibition. The PROTAC cocktail superiorly abrogates the expression of stem cell markers c‐Myc, NANOG, STATA5A, and FOXM1 (Figure 4K).

### 2.5 The AURKA PROTAC Cocktail Relieves the Hook Effect

**Figure 5:**
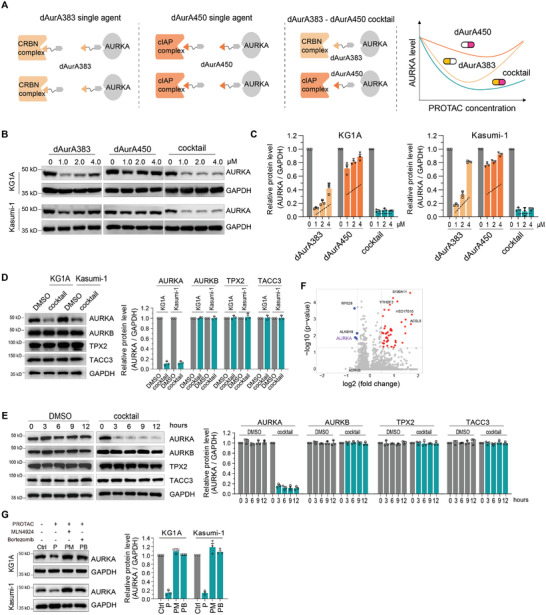
An AURKA PROTAC cocktail relieves the hook effect. A) Scheme depicting the strategy to combine dAurA383 and dAurA450 to relieve the hook effect. B) Degradation of endogenous AURKA in KG1A and Kasumi‐1 cells following 12 h treatment with the indicated concentration of PROTACs. C) Relative AURKA protein levels in panel B were quantified using the ImageJ software. D) KG1A and Kasumi‐1 cells were treated with PROTAC cocktail (4 × 10^−6^ m) for 12 h. The expression of AURKA and related proteins were detected. E) KG1A cells were arrested with nocodazole (0.1 µg mL^−1^) for 16 h followed by PROTAC cocktail (4 × 10^−6^ m) treatment for indicated times. The expression of AURKA and related proteins were detected. F) TMT‐based quantitative proteomics after treatment with dAurA383 and dAurA450 cocktail (1 × 10^−6^ m) or the DMSO Vehicle for 6 h in KG1A cells. The differentially expressed proteins are presented in the volcano plot. G) Degradation of endogenous AURKA in KG1A and Kasumi‐1 cells following 12 h treatment with PROTAC cocktail (4 × 10^−6^ m), NEDD8‐activating enzyme inhibitor MLN4924 (1 × 10^−6^ m) or proteasomal inhibitor Bortezomib (25 × 10^−9^ m).

The hook effect is a phenomenon manifesting the loss of degradation activity of PROTACs at high concentrations.^[^ ^51^ ^]^ The redundant PROTACs generate unproductive binary complexes, which compete for formation of the functional ternary complexes (**Figure** **5**A, left two panels). We assume that the PROTAC cocktail could relieve the hook effect via expanding the available E3 ligase pools (Figure 5A, right two panels). Indeed, the single PROTACs display hook effect at the concentration of 2 × 10^−6^ m, whereas the PROTAC cocktail displays hook effect at the concentration of 8 × 10^−6^ m in KG1A and Kasumi‐1 cells (Figure 5B,C and S6A‐B). The remission of hook effect by cocktail is also observed in HL60 and U937 cells (Figure S6C,D, Supporting Information).

We further examined the specificity of the PROTAC cocktail. The treatment of PROTAC cocktail does not reduce the expression of AURKB, TPX2, and TACC3 in both proliferating and mitotic arrest cells (Figure 5D,E). The TMT‐based quantitative proteomic analysis confirms the specific degradation of AURKA by PROTAC cocktail (Figure 5F). The degradation activity of PROTAC cocktail is blocked by MLN4924 and Bortezomib (Figure 5G).

### 2.6 The AURKA PROTAC Cocktail Induces AML Regression in a Xenograft Mouse Model and Primary AML Blasts

**Figure 6:**
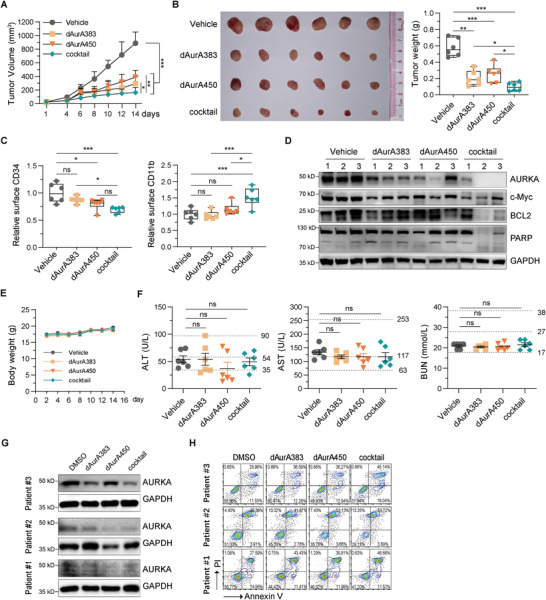
AURKA PROTAC cocktail induces AML regression in a xenograft mouse model and primary blasts. A) Nude mice bearing KG1A xenografts were intraperitoneally injected with dAurA383, dAurA450, and cocktail (30 µmol kg^−1^ day^−1^). The tumor volume from 1 to 14 days is plotted versus time. B) Left panel, tumors resected from the mice in each group are shown. Right panel: statistical analysis of the tumor weight. C) The surface CD34 and CD11b of dissected tumors were analyzed by FACS assays. D) The indicated proteins of dissected tumors were detected by Western blot. E) The body weight of the mice were measured and plotted against time. F) Plasma ALT, AST, and BUN of the mice were measured. The dotted lines display the normal reference values of BALB/c nude mice in Charles River Laboratories. G) AURKA expression was detected by Western blot in AML primary blasts treated with AURKA PROTACs (1 × 10^−6^ m) for 6 h. H) Cell apoptosis was analyzed in AML primary blasts treated with AURKA PROTACs (1 × 10^−6^ m) for 48 h. Statistics, significance: one‐way ANOVA with Bonferroni correction (A,B,C,F); ns, not significant; *P < 0.05; **P < 0.01; ***P < 0.001.

We evaluated the in vivo anticancer effects of AURKA PROTAC cocktail in a xenograft model using MLN8237 as a positive control. Nude mice harboring KG1A xenografts were subjected to dAurA383, dAurA450, or the PROTAC cocktail by daily intraperitoneal injection. As shown in **Figure** **6**A,B and Figure S7A,B (Supporting Information), at the dose level of 30 µmol kg^−1^ day^−1^, AURKA PROTAC cocktail significantly suppresses KG1A cell growth in vivo. We further isolated the KG1A cells from the dissected tumor tissues. AURKA PROTAC cocktail decreases the expression of hematopoietic stem/progenitor cell marker CD34 and increases the expression of myeloid‐monocytic lineage differentiation marker CD11b^[^ ^52^ ^]^ (Figure 6C). Meanwhile, AURKA PROTAC cocktail more effectively degrades AURKA and induces cancer cell apoptosis (Figure 6D). The body weights show no significant differences between the PROTAC treated and the vehicle treated mice (Figure 6E), while show a slight decrease in MLN8237 treated mice (Figure S7C, Supporting Information). Additionally, the plasma alanine aminotransferase (ALT), aspartate aminotransferase (AST), and blood urea nitrogen (BUN) in the PROTAC cocktail treatment group are not statistically different from that in vehicle control group (Figure 6F).

Finally, we assessed the inhibition effect of the AURKA PROTAC cocktail in malignant bone marrow mononuclear cells (BMMCs) obtained by gradient centrifuged from three pathologically confirmed de novo AML patients. Western blot analysis shows AURKA PROTAC cocktail effectively degrades AURKA protein in AML patient derived BMMCs (Figure 6G). The AURKA PROTAC cocktail significantly induces apoptosis of patient derived AML blasts (Figure 6H).

## 3 Discussion

**Figure 7:**
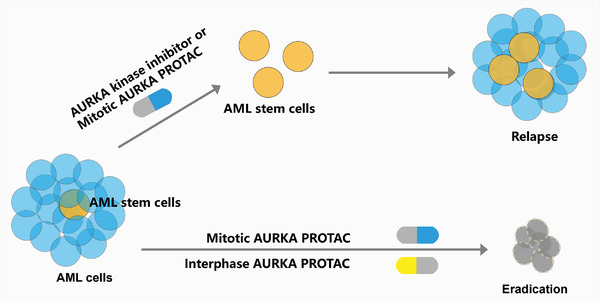
Graphic depiction of the working model of AURKA PROTAC cocktail. AURKA kinase inhibitor or the mitotic AURKA PROTAC preferentially inhibits or degrades mitotic kinase‐dependent AURKA and kills the proliferative AML cells, while the interphase AURKA PROTAC preferentially degrades the interphase kinase‐independent AURKA and attenuates cancer stemness. The PROTAC cocktail eliminates both the proliferative AML cells and AML stem cells.

PROTAC is an emerging drug discovery paradigm.^[^ ^53^ ^]^ In recent years, researchers have focused on developing new compounds to degrade various target proteins such as kinases,^[^ ^54^ ^]^ transcription factors,^[^ ^55^, ^56^, ^57^ ^]^ and “undruggable” proteins.^[^ ^58^ ^]^ However, the spatial‐temporal distribution and multifaceted functions of target proteins have hindered the effectiveness of the first generation PROTACs. In this study, we have developed a set of AURKA PROTACs based on different E3 ligases. The CRBN‐based dAurA383 preferentially targets canonical functions of mitotic AURKA, while cIAP‐based dAurA450 preferentially targets the emerging noncanonical functions of interphase AURKA. We conclude that the dAurA383 and dAur450 cocktail would both eliminate the proliferative AML cells and abrogate AML stem cells (**Figure** **7**). The spatial‐temporal drug administration strategy was validated as a feasible approach in animal xenografts and the patient derived primary blasts. Based on our findings, we propose a PROTAC cocktail strategy as a second generation of PROTACs to overcome the multifaceted oncogenic functions of targets.

The proteomic and transcriptomic analyses support the cell cycle phase specific functions of dAurA383 and dAurA450. Fox example, dAurA383 treatment triggers the mitotic related processes such as “mitotic cell cycle,” echoing that dAurA383 arrests AML cells at mitosis by removing the spindle pool of AURKA, decreasing the phosphorylation of TACC3 and disrupting spindle microtubules.^[^ ^59^ ^]^ However, dAurA383 treatment induces stemness related processes such as “CONRAD stem cell,” which is consistent with our finding that dAurA383 induces a population of CD34 high stem cells. dAurA450 treatment suppresses the stemness related processes such as “hallmark MYC/E2F targets,” “EPPERT Progenitor,” “WONG Embryonic stem cell core,” and “Oxidative phosphorylation,” supporting previous studies that interphase AURKA facilitates stem‐cell‐like phenotypes^[^ ^22^, ^23^, ^24^ ^]^ and increases energy production,^[^ ^45^, ^46^ ^]^ and in line with that dAurA450 induces CD34 low cells and suppresses the expression of stem cell markers including c‐Myc, NANOG, STAT5A, and FOXM1. The stemness inhibition may also explain the result that dAurA450 delays the cell division but not obviously changes the cell cycle phase distribution.

Besides targeting the multifunctions of AURKA, another advantage of PROTAC cocktail is reliving the hook effect, a phenomenon manifesting the loss of degradation activity of PROTACs at higher concentrations.^[^ ^51^ ^]^ PROTAC overdose generates saturated individual [E3 ligase‐PROTAC] and [PROTAC‐target protein] binary complexes which compete for the formation of the [E3 ligase‐PROTAC‐target protein] ternary complex.^[^ ^60^ ^]^ Employing more available E3 ligases partially explains how the cocktail alleviates the hook effect. Other mechanisms by which the different E3 ligase‐based PROTACs degrade AURKA levels in different degrees and how PROTAC cocktail synergistically degrades AURKA through the ubiquitin‐proteasome system warrant further investigations. Theoretically, all PROTAC molecules exhibit the “hook effect” at high concentrations. Considering the differences of drug bioavailability including absorption, metabolism, and excretion, the effective dose range of single PROTACs and cocktail to degrade AURKA varies in different cells. Nevertheless, the cocktail strategy might expand the therapeutic window of PROTACs.


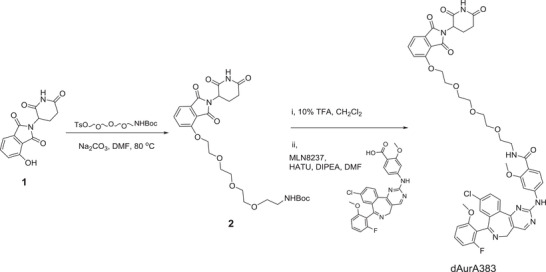


The E3 ligase ligands binding to CRBN, cIAP, VHL, and MDM2 are frequently used to design PROTAC degraders. However, how to choose the E3 ligand rationally is an unsettled issue in PROTAC design. We finally obtained 5 CRBN‐based PROTACs, 3 VHL‐based PROTACs, and 4 cIAP‐based PROTACs with the commonly used E3 ligands^[^ ^61^ ^]^ and different linkers. Generally, the CRBN‐based PROTACs have better degradation activity on AURKA. With similar linker length, CRBN‐based dAurA383, VHL‐based dAurA1067, and cIAP‐based dAurA1072 exhibit disparate degradation activity, suggesting the predominant role of E3 ligase in PROTAC activity. Besides, the degradation activity of PROTACs increases with the extension of the linker length (from 6 atoms to 15 atoms) in CRBN‐based group, which is in line with the published finding that linker length is a critical parameter in PROTAC activity.^[^ ^34^ ^]^ Meanwhile, all of the reported CRBN‐based AURKA PROTACs (dAurA383 with a 12‐atom linker, dAurA1071 with a 15‐atom linker, PROTAC‐D with a 15‐atom linker,^[^ ^34^ ^]^ PROTAC‐DX with a 21‐atom linker,^[^ ^34^ ^]^ and JB170 with a 12‐atom linker^[^ ^33^ ^]^) with more than about 11 atoms can effectively degrade AURKA, which implies when the linker reaches a certain length, the benefit from further linker extension is limited. By summary, the correlation between the expression of E3 ligases and the distribution of target protein in cell cycle deserves the preferential consideration in choosing E3 ligand to design PROTACs. Besides, the physical properties of linkers such as linker length are also crucial for PROTAC activity.

Although all of dAurA383, dAurA425, and dAurA450 effectively bind to AURKA as indicated by the SPR and SPPIER assays, only the CRBN‐based dAurA383 degrades the vast majority of AURKA. By blocking the ubiquitin‐proteasome pathway including suppression of NEDD8‐activating enzyme E1, knockdown of E3 ligases, supplement with E3 ligands, and inhibition of proteasome, we proved that dAurA425 and dAurA450 also induce AURKA degradation through the ubiquitin‐proteasome system. The distribution of E3 ligases in cell cycle contributes to the inadequate degradation efficiency of dAurA425 and dAurA450. Other reasons for the low efficiency of dAurA425 and dAurA450 such as the “degron” motifs^[^ ^62^ ^]^ recognized by VHL and cIAP1 within AURKA need to be further investigated.

Taken together, this study is the first to describe a PROTAC combination strategy to overcome the multifaceted oncogenic functions of AURKA and relive the hook effect of PROTACs. The concept of the secondary generation PROTAC cocktail is worthy to be tested on more targets in future.

## 4 Experimental Section

### Synthesis of PROTAC dAurA383, dAurA425, and dAurA450

AURKA PROTACs were designed by the laboratory and synthesized from MLN8237 and the derived ligands bound to one of E3 ubiquitin ligases CRBN, VHL, and cIAP. AURKA PROTACs were purified by silica gel column chromatography and resolved in DMSO as a stock solution (10 × 10^−3^ m) at −20 °C. They are abbreviated as dAurA379, dAurA380, dAurA383, dAurA393, dAurA408, dAurA425, dAurA448, dAurA449, dAurA450, dAurA1067, dAurA1071, and dAurA1072 (Figure 1B; Figure S1B, Supporting Information). Their synthesis is exemplified by compounds dAurA383, dAurA425, and dAurA450. The rest PROTACS are synthesized using similar schemes as given in the Supporting Information.

MLN8237 (GLPBIO, Cat# GC12690) and compounds **1–7** were purchased from commercial vendors or prepared following the reported works.^[^ ^63^, ^64^ ^]^ In this study, all the biologically tested compounds were >95% pure by HPLC analysis. ^1^H NMR (400 or 500 MHz) and ^13^C NMR (101 or 126 MHz) spectra were obtained by Bruker Avance spectrometer 400/500. Chemical shifts are given in ppm (*δ*) that internally referenced to CDCl_3_ with 7.26 for ^1^H and 77.16 for ^13^C, *d*_6_‐DMSO with 2.50 for ^1^H and 39.5 for ^13^C, and *D*_4_‐MeOD with 3.31 for ^1^H and 49.0 for ^13^C. Multiplicity of ^1^H NMR signals was reported as follows: s, singlet; d, doublet; t, triplet; q, quartet; m, multiplet. HPLC analysis was performed on an Agilent 1260 with a C18 column (4.6 mm × 150 mm, 1.8 µm) with the flow rate 1 mL min^−1^. High‐resolution mass spectra were conducted by Thermo Fisher QExactive. The progress of the reactions was monitored by thin‐layer chromatography on a glass plate coated with silica gel with fluorescent indicator (GF254). Column chromatography was performed on silica gel (200–300 mesh).

### Synthesis of dAurA383

Compound **2** was prepared following the previous report.^[^ ^63^ ^]^ After cleavage of Boc in compound **2** under 10% TFA solution in dichloromethane, to a solution of the freshly prepared free amine (35.0 mg) in DMF (1.0 mL) was added DIPEA (63 *μ*L), MLN8237 (40.0 mg), and HATU (29.3 mg). The reaction mixture was stirred for 2 h at room temperature. EtOAc was added, and the mixture washed with water three times. The organic layer was dried over MgSO_4_, filtered, and evaporated in vacuo. The resulting residue was purified by silica column chromatography (MeOH/CH_2_Cl_2_ = 1/20) to provide dAurA383 as a yellow solid (39 mg, 52%). ^1^H NMR (400 MHz, CDCl_3_) *δ* 10.59 (s, 1H), 8.62 (brs, 1H), 8.53 (s, 1H), 8.25‐8.22 (m, 2H), 8.14 (d, *J* = 8.7 Hz, 1H), 7.69‐7.54 (m, 3H), 7.45 (d, *J* = 7.3 Hz, 1H), 7.35 (s, 1H), 7.34‐7.27 (m, 3H), 6.76 (m, 2H), 5.00 (dd, *J* = 12.5, 5.4 Hz, 1H), 4.39‐4.31 (m, 2H), 3.77 (s, 3H), 3.73 (m, 2H), 3.69‐3.65 (m, 10H), 3.50‐3.20 (m, 2H), 2.90 (m, 2H), 2.76 (m, 1H), 2.20‐2.09 (m, 1H) . ^13^C NMR (101 MHz, CDCl_3_) *δ* 171.7, 170.2, 167.2, 165.9, 165.4, 159.2, 158.4, 156.5, 156.4, 144.4, 138.1, 136.6, 136.0, 135.3, 133.8, 133.1, 131.1, 130.9, 130.5, 130.3, 123.6, 119.6, 117.2, 116.2, 114.8, 110.7, 108.5, 108.3, 107.1, 101.4, 771.2, 70.8, 70.6, 70.2, 69.7, 69.4, 56.2, 55.6, 50.4, 49.4, 45.9, 39.5, 31.6, 22.7. HRMS (*m*/*z*, ESI) for C_48_H_46_O_11_N_7_ClF (M+H^+^): calcd 950.29224; found 950.29195. HPLC purity: 95.99%.

### Synthesis of dAurA425


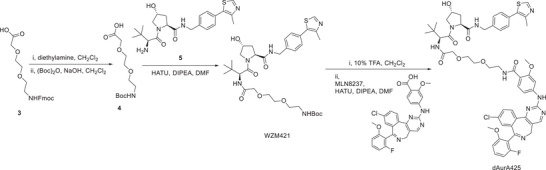



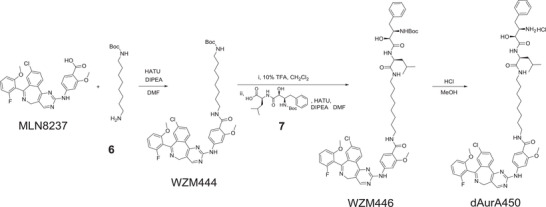


Compounds **4** and **5** were prepared following the previous report.^[^ ^63^ ^]^ To a solution of **5** (40.0 mg) in DMF (1.0 mL) was added DIPEA (77.0 *μ*L), **4** (29.4 mg), and HATU (35.3 mg). The reaction solution was stirred for 2 h at room temperature. EtOAc was added, and the mixture washed with water three times. The organic layer was dried over MgSO_4_, filtered, and evaporated in vacuo. The resulting residue was purified by column chromatography (MeOH/CH_2_Cl_2_ = 1/20) to provide WZM421 as a yellow solid (20.0 mg, 34%).

TFA (100 *μ*L) was added to a solution of WZM421 (20 mg) in dichloromethane (1 mL). The reaction mixture was stirred for 3 h at room temperature, and then it was evaporated in vacuo to provide Boc‐cleaved free amine that was used directly for the next step without purification. To a solution of the freshly prepared free amine in DMF (1.0 mL) was added DIPEA (17.2 *μ*L), MLN8237 (18.0 mg), and HATU (13.2 mg). The reaction solution was stirred for 2 h at room temperature. EtOAc was added, and the mixture washed with water three times. The organic layer was dried over MgSO_4_, filtered, and evaporated in vacuo. The resulting residue was purified by column chromatography (MeOH/CH_2_Cl_2_ = 1/20) to provide dAurA425 as a yellow solid (5.0 mg, 13%). ^1^H NMR (500 MHz, CDCl_3_) *δ* 8.66 (s, 1H), 8.51 (s, 1H), 8.20 (m, 2H), 8.09 (d, *J* = 8.2 Hz, 1H), 7.88 (s, 1H), 7.57 (d, *J* = 8.4 Hz, 1H), 7.53 (s, 1H), 7.41 (d, *J* = 8.4 Hz, 1H), 7.37‐7.27 (m, 6H), 7.05 (d, *J* = 7.3 Hz, 1H), 6.79 (m, 2H), 4.87 (brs, 1H), 4.60‐4.45 (m, 4H), 4.28 (m, 1H), 4.07‐4.00 (m, 4H), 3.93 (s, 3H), 3.72‐3.65 (m, 7H), 3.57 (d, *J* = 10.2 Hz, 1H), 3.23‐3.19 (m, 3H), 2.47 (m, 3H), 2.04 (t, *J* = 10.6 Hz, 1H), 1.39 (t, *J* = 7.2 Hz, 3H), 0.92 (s, 9H). ^13^C NMR (126 MHz, CDCl_3_) *δ* 171.2, 1710, 170.4, 165.7, 159.4, 158.7, 156.8, 150.4, 148.5, 148.0, 144.0, 138.3, 136.2, 136.2, 135.2, 132.9, 131.8, 131.1, 131.0, 131.0, 130.40, 130.4, 130.30, 129.6, 128.7, 128.3, 124.1, 115.4, 111.0, 107.4, 107.2, 107.2, 101.6, 100.2, 81.6, 71.6, 70.5, 70.5, 70.2, 58.6, 57.2, 56.9, 56.0, 50.5, 43.3, 39.6, 36.2, 35.6, 26.5, 16.2. HRMS (*m*/*z*, ESI) for C_55_H_60_O_9_N_9_ClFS (M + H^+^): calcd1076.39018, found1076.39003. HPLC purity 99.73%.

### Synthesis of dAurA450

DIPEA (34.1 *μ*L), MLN8237 (40.0 mg), and HATU (29.3 mg) were added to a solution of compound **6** (17.8 mg) in DMF (0.5 mL). The reaction solution was stirred for 2 h at room temperature. EtOAc was added, and the mixture washed with water three times. The organic layer was dried over MgSO_4_, filtered, and evaporated in vacuo. The resulting residue was purified by column chromatography (MeOH/CH_2_Cl_2_ = 1/20) to provide WZM444 as a yellow solid (55.0 mg, 96%).

TFA (100 *μ*L) was added to a solution of WZM444 (55.0 mg) in dichloromethane (1 mL). The reaction solution was stirred for 3 h at room temperature before it was concentrated in vacuo to provide free amine. To a solution of the freshly prepared free amine (47.0 mg) in DMF (0.5 mL) was added DIPEA (33.0 *μ*L), compound **7** (36.5 mg), and HATU (34.0 mg). The reaction solution was stirred for 2 h at room temperature. EtOAc was added, and the mixture was washed with water three times. The organic layer was dried over MgSO_4_, filtered, and evaporated in vacuo. The resulting residue was purified by column chromatography (MeOH/CH_2_Cl_2_ = 1/20) to provide WZM446 as a yellow solid (32.0 mg, 42%). ^1^H NMR (500 MHz, CDCl_3_) *δ* 8.48(s, 1H), 8.22 (d, *J* = 8.5 Hz, 1H), 8.14 (d, *J* = 8.5 Hz, 1H), 7.96 (s, 1H), 7.85 (m, 1H), 7.71 (s, 1H), 7.56 (dd, *J* = 8.5, 2.0 Hz, 1H), 7.32 (m, 1H), 7.29 (d, *J* = 6.7 Hz, 1H), 7.24‐7.21 (m, 4H), 7.18 (d, *J* = 6.8 Hz, 1H), 7.14 (d, *J* = 8.4 Hz, 1H), 6.74 (brs, 2H), 6.52 (s, 1H), 5.88 (d, *J* = 6.2 Hz, 1H), 5.16 (d, *J* = 8.4 Hz, 1H), 4.49‐4.36 (m, 1H), 4.15‐4.00(m, 2H), 3.98 (s, 3H), 3.42 (m, 3H), 3.24 (m, 2H), 3.11‐2.99 (m, 3H), 1.81 (m, 3H), 1.69 (m, 1H), 1.60‐1.55 (m, 4H), 1.45‐1.42 (m, 3H), 1.36 (s, 9H), 1.30‐1.25 (m, 4H), 0.91 (d, *J* = 6.0 Hz, 3H), 0.88 (d, *J* = 6.0 Hz, 3H). LCMS (*m/z*, ESI): 1035.3 (M + H^+^).

WZM446 (6.20 mg) was added to HCl solution in MeOH (1 m, 1 mL), and the reaction solution was stirred for 5 h at room temperature. The solution was evaporated in vacuo to provide dAurA450 as a yellow solid (4.90 mg, 100%). ^1^H NMR (400 MHz, MeOD) *δ* 8.64 (s, 1H), 8.42 (d, *J* = 8.5 Hz, 1H), 8.00 (d, *J* = 1.7 Hz, 1H), 7.92 (d, *J* = 8.7 Hz, 1H), 7.83 (dd, *J* = 8.5, 2.1 Hz, 1H), 7.53 (dd, *J* = 15.2, 8.5 Hz, 1H),7.40‐2.7 (m, 7H), 6.90 (brs, 2H), 4.35 (dd, *J* = 8.3, 6.4 Hz, 1H), 4.12 (d, *J* = 3.1 Hz, 1H), 4.01 (s, 3H), 3.78 (m, 1H), 3.40 (t, *J* = 7.1 Hz, 2H), 3.19‐3.08 (m, 3H), 2.92 (m, 1H), 1.65‐1.58 (m, 3H), 1.48 (m, 2H), 1.40‐ 1.30 (m, 10H), 0.97 (d, *J* = 10.8 Hz, 3H), 0.95 (d, *J* = 10.8 Hz, 3H). HRMS (*m*/*z*, ESI) for C_51_H_61_O_6_N_8_ClF (M + H^+^): calcd 935.43811, found 935.43822. HPLC purity: 96.88%.

### SPR Analysis

The binding of MLN8237, AURKA PROTACs or E3 binding ligands with AURKA was detected by the intermolecular interaction and analysis system Biacore T200. In brief, chemicals and AURKA proteins were prepared. Homemade AURKA protein was previously purified and embedded on a CM5 microarray. Chemicals MLN8237, dAurA383, dAurA425, dAurA450, and E3 binding ligands were serial diluted to 50, 25, 12.5, 6.25, 3.12, 1.56 × 10^−6^ m. After Equipment debugging, the microarray was successively flowed over different concentrations of small molecules MLN8237, AURKA PROTACs or E3 binding ligands to determine the binding kinetics and affinity with AURKA proteins.

### Cell Lines and Culture Conditions

Human leukemia cell lines HL60, U937, and NB4 were obtained from the laboratory repertory and cultured in Roswell Park Memorial Institute (RPMI) 1640 (C11875500BT, Gibco) containing 10% fetal bovine serum (FBS, 10270106, Gibco). Human leukemia cell lines KG1a and Kasumi‐1 were donated by Prof. Zijie Long (The Third Affiliated Hospital of Sun Yat‐sen University) and cultured in RPMI 1640 containing 20% FBS. Human leukemia cell lines THP1 were donated by Prof. Xiaofeng Zhu of Sun Yat‐sen University Cancer Center and cultured in RPMI 1640 containing 10% heat‐inactivated FBS (30 min at 56 °C) and 55 × 10^−6^ m 2‐mercaptoethanol (21985‐023, Gibco). Human embryonic kidney cell line HEK293T was from the laboratory repertory and cultured in Dulbecco's modified Eagle medium (C11995500BT, Gibco) containing 10% FBS. Cell lines were cultured in a constant humidity incubator containing 5% CO_2_ at 37 °C and were mycoplasma free.

### Antibodies

Antibodies against GAPDH (ET1601‐4, Huabio), AURKA (14475S, Cell Signaling Technology), PARP (9532S, Cell Signaling Technology), c‐Myc (AF6513, Beyotime), pH3S10 (9701S, Cell Signaling Technology), H3 (4499, Cell Signaling Technology), Bcl‐2 (15071, Cell Signaling Technology), CRBN (AF6564, Beyotime), cIAP1 (GTX110087, GeneTex), VHL (68547, CST), cyclin E1 (AF2491, Beyotime), cyclin B1 (AF1606, Beyotime), STAT5A (AF2038, Beyotime), NANOG (ab203919, Abcam), FOXM1 (AF6924, Beyotime), AURKB (GTX132702, GeneTex), TACC3 (AF1345, Beyotime), TPX2 (GTX115654, GeneTex), Phospho‐TACC3 (Ser558) (AF4506, Affinity Biosciences), and Phospho‐Aurora A (Thr288)/Aurora B (Thr232)/Aurora C (Thr198) (2914S, CST) were used for the Western blot analysis. PE antihuman CD34 (343506, Biolegend) and Alexa Fluor 700 antimouse/human CD11b (101222, Biolegend) were used for the flow cytometry analysis. AURKA (14475S, Cell Signaling Technology), *α*‐Tubulin (AT819, Beyotime), Alexa Fluor 488 goat antirabbit IgG (H+L) (A11034, Invitrogen), Alexa Fluor 546 goat antimouse IgG (H+L) (A11003, Invitrogen) were used for immunofluorescence assay.

### Plasmid Construction

Teton‐pLKO plasmids were constructed to knockdown CRBN, VHL, and cIAP1 under doxycycline (HY‐N0565B, MCE) treatment. In brief, oligos targeting CRBN, VHL or cIAP1 were synthesized by the company. Oligos were annealed in the annealing buffer (100 × 10^−3^ m Tris‐HCl, 1 m NaCl, pH = 7.4) and inserted into AgeI and EcoRI digested Teton‐pLKO plasmid with T4 DNA ligase to construct Teton‐pLKO‐shCRBN, Teton‐pLKO‐shVHL, and Teton‐pLKO‐shcIAP1. shRNA sequences are shown in Table S3 of the Supporting Information.

Overexpression plasmids were constructed for the SPPIER assay. In brief, the cDNA of CRBN, VHL, and cIAP1 was cloned from the homemade cDNA library, while the sequences of HOTag3 and Hotag6 were synthesized by the company. The cDNA of CRBN, VHL or cIAP1 was cloned into pLVX‐EGFP with HOTag6 as CRBN‐EGFP‐HOTag6, VHL‐EGFP‐HOTag6, and cIAP1‐EGFP‐HOTag6, while the cDNA of HOTag3 was cloned into pLVX‐AURKA‐mCherry as AURKA‐mCherry‐HOTag3.

### Lentivirus Packaging and Transfection

For transient transfection, HEK293T cells were cultured in an antibiotic‐free medium at 60% convergence. Plasmids containing Opti‐MEM (31985070, Gibco) was gently mixed and incubated with Lipofectamine 2000 (11668019, Invitrogen) containing Opti‐MEM at room temperature for 20 min. The mixture was dropped into HEK293T cells for indicated time. The transfected cells were used for functional experiments.

For stable transfection, lentivirus was first produced with HEK293T cells cotransfected with the lentiviral constructs, packaging plasmid (psPAX2) and envelope plasmid (pMD2.G) using Lipofectamine 2000 with the ratio 4:3:1. Virus was collected, filtered, and added to KG1A cells with polybrene (HY‐112735, MCE) for 24 h. The infected KG1A cells were further selected with 1 µg mL^−1^ puromycin (0219453925, MP) for 2 weeks to get stable transfected cell lines.

### SPPIER Assay

To detect PROTAC‐induced ternary complexes formation, the EGFP‐based SPPIER assays^[^ ^42^, ^43^ ^]^ to DF‐SPPIER was optimized by mCherry to label AURKA (AURKA‐mCherry‐HOTag3) and EGFP to label E3 ubiquitin ligase (E3 ubiquitin ligase‐EGFP‐HOTag6). HEK293T cells in 35 mm glass‐bottom dishes (801002, NEST) were transiently cotransfected with 1.0 µg AURKA‐mCherry‐HOTag3 together with 1.0 µg CRBN‐EGFP‐HOTag6, VHL‐EGFP‐HOTag6 or cIAP1‐EGFP‐HOTag6 in combination for 12 h and supplied with DMSO, AURKA PROTACs or E3 ligands (500 × 10^−9^ m) for 3 h. Cells were fixed with 4% paraformaldehyde (BL539A, Biosharp) and stained with DAPI (D9542, Sigma) for immunofluorescence analysis. Images were acquired with LSM880 system (Zeiss, Germany). The ZEN software (Zeiss, Germany) was used to process the images including export the yellow droplet images. The profile of red and green fluorescence intensity was analyzed using plot profile function in ImageJ. The sum of yellow droplets pixel fluorescence intensity was analyzed using analyzed particle function in ImageJ.

### Cell Growth Assay

Cell proliferation and survival were detected by CCK‐8 assay. In brief, the leukemia cells were dispersed into a 96‐well plate at a density of about 5 × 10^3^ cells per well, and treated with the indicated concentration of drugs for an indicated time. At the end of experiment, 10 µL of CCK8 reagent (40203ES80, Yeasen) was added to each well and incubated at 37 °C for 2 h. The absorbance was determined at a test wavelength of 450 nm and the relative proliferation or survival of cells was calculated. The IC_50_ value was calculated using GraphPad Prism 8 (Inc., LaJolla, CA, USA).

### Cell Synchronization

Cells were synchronized at the G1/S boundary by the standard double thymidine blocking method. Briefly, cells were first blocked with thymidine (HY‐N1150, MCE, 2 × 10^−3^ m) for 16 h, washed with phosphate buffered saline (PBS, G0002, Servicebio) twice, released in fresh media for 9 h, and then exposed to thymidine (2 × 10^−3^ m) for an additional 16 h. Synchronized cells were released into fresh media and collected at the indicated time points for cell cycle and Western blot analysis.

### Cell Cycle Analysis

Flow cytometry was used to determine the cell cycle phase distribution. Cells were collected, centrifuged at 800 RPM for 5 min, washed with PBS, and then fixed in 70% ethanol at 4 °C overnight. Subsequently, the cells were washed and resuspended in PBS staining solution containing 50 µg mL^−1^ propidium iodide (81845, Sigma), 100 µg mL^−1^ RNase A (10109169001, Roche), 0.2% Triton X‐100 (10789704001, Roche), and incubated at 37 °C for 30 min in the dark. The cells were then washed, resuspended with 200 µL PBS and detected with flow cytometry under the PI/PE channel. The cell cycle phase distribution was analyzed using FlowJo software.

### CFSE Staining Assay

Briefly, cells were prestained with CFSE probe (S8269, Selleck, 0.5 × 10^−6^ m) in a serum free medium for 15 min, washed with PBS and then suspended and cultured in fresh medium with indicated concentrations of AURKA PROTACs for 72 h. Then, cells were collected, washed, and resuspended in 250 µL PBS. CFSE fluorescence was detected by flow cytometry under the FITC channel. The relative mean fluorescence intensity (MFI) of CFSE were analyzed by CytExpert and FlowJo software.

### Apoptosis Analysis

The effect of AURKA PROTACs on apoptosis of leukemia cell lines and primary blasts were detected by flow cytometry according to the manufacturer's protocol with some modifications. Primary blasts were enriched with Ficoll‐Hypaque solution (LTS1077, TBD) by density gradient centrifugation and then washed and counted for further treatment. AURKA PROTACs treated leukemia cells or primary blasts were collected, centrifuged at 800 RPM for 5 min, and washed with PBS. For Annexin V assays, Annexin V‐AF647/PI apoptosis kit (AP006, YiShan Biotech) was used. The cells were resuspended in a 100 µL 1×Annexin V binding buffer with 5 µL Annexin V‐647 and 10 µL PI and incubated at room temperature for 15 min in the dark. Then, 400 µL 1×Annexin V binding buffer was added into each sample and Annexin V positive cells were detected by flow cytometry. The results were analyzed by CytExpert software.

### Methylcellulose‐Based Colony Forming Cell Assay

The clonogenic activity of leukemia stem cells was determined by methylcellulose‐based CFC assay. For the primary colony formation, leukemia cells were pretreated with indicated concentration of AURKA PROTACs or DMSO for 12 h and then counted and uniformly resuspended in a 250 µL cell culture medium containing twice the concentration of indicated drugs at a density of 500 cells per well. The mixture was then mixed with an equal volume of 1% methyl cellulose (M0512, Sigma) and inoculated into 24‐well cell culture plates to culture. After 14 days, images were acquired with microscope, while the number and size of colonies formed in each treatment were counted. The secondary colony formation was conducted with the same procedure using cells collected from primary colonies.

### Western Blot Analysis

PROTACs, E3 ligands, MLN8237 (S1133, Selleck), MLN4924 (B1036‐5.1, APExBIO), and Bortezomib (T2399, TargetMol) treated samples as well as released synchronized cells were subjected for immunoblot analysis. Whole cell lysates were extracted using standard Western blot procedures. Fresh cells were lysed on ice in an RIPA buffer (50 × 10^−3^ m Tris pH = 8.0, 1 × 10^−3^ m EDTA, 150 × 10^−3^ m NaCl, 1% NP‐40, 0.5% sodium deoxycholic acid, 0.1% SDS) containing phosphatase and protease inhibitors (C0002 and C0001, TargetMol) for 15 min. Ultrasound was performed at 80 Hz for 5 min and the impurities were removed by centrifuge at 12 000 RPM for 10 min. The concentration of protein supernatant was determined by the Bradford method and an equal amount of protein was subjected for electrophoresis using the procedure of 60V for 30 min followed by 120 V for 1 h. The protein was transferred onto a PVDF membrane with 300 mA for 90 min. After being blocked at room temperature for 1 h with 5% bovine serum albumin (BSA, FC0077, MP), the membrane was incubated overnight with the indicated antibodies at 4 °C. Subsequently, the PVDF membrane was washed three times with TBST and incubated with the peroxidase‐conjugated secondary antibody for 1 h at room temperature. Protein expression was monitored by the BioRad chemiluminescent imaging system with an enhanced chemiluminescence reagent (SQ201, EpiZyme).

### Immunofluorescence

KG1A cells arrested with nocodazole (0.1 µg mL^−1^) for 16 h were treated with indicated concentration of PROTACs, MLN8237 or DMSO for 6 h. Cells were collected, washed and resuspended with PBS and centrifuged onto glass slides. Cells were then fixed with 4% paraformaldehyde (BL539A, Biosharp) for 10 min, permeabilized with 0.1% Triton X‐100 for 5 min and washed with PBS. After blocking with 3% BSA, cells were incubated with the primary antibodies at 4 °C overnight, washed and incubated with secondary antibody at room temperature for 1 h followed by DAPI staining (D9542, Sigma‐Aldrich) and washing step. Finally, the samples were sealed with antifade mounting medium (P0126, Beyotime) and coverslips. The images were taken with laser scanning confocal microscope.

The primary antibodies used were AURKA rabbit mAb (1:200, CST, 14475S) and *α*‐Tubulin mouse mAb (1:200, AT819, Beyotime). The secondary antibodies used were Alexa Fluor 488 goat antirabbit IgG (H+L) (1:200, A11034, Invitrogen) and Alexa Fluor 546 goat antimouse IgG (H+L) (1:200, A11003, Invitrogen).

### Real‐Time PCR

Total RNA was extracted from leukemia cells using the HiPure Total RNA Plus Mini Kit (R4121‐02, Magen). Using total RNA as a template, reverse transcription was conducted and cDNA was synthesized with TransScript All‐in‐one first‐strand cDNA Synthesis SuperMix for qPCR (AT341‐01, TransGen). According to the instructions of ChamQ Universal SYBR qPCR Master Mix (Q311‐03, Vazyme), qPCR was performed using a Bio‐RAD CFX96 fluorescence quantitative PCR instrument and the relative expression of the interested genes was calculated by the 2ˆ(ΔΔCt) method. Primers were designed according to the PrimerBank website (https://pga.mgh.harvard.edu/primerbank/). qPCR primers are shown in Table S4 of the Supporting Information.

### Evaluation of Drug Interactions–Combination Index (CI) Calculation

The CompuSyn software (CompuSyn, Paramus, NJ, USA) was used to evaluate the CI of cells that responded to the drugs in combination. In brief, the percentage of restrained cells upon a series concentration of single PROTACs or cocktail was acquired from the CCK‐8 assay and subjected to CompuSyn software. CI values below 0.8, between 0.8 and 1.2, and over 1.2 were defined as synergistic, additive, and antagonistic, respectively. The relative cell survival and CI values of each treatment were plotted.

### Xenograft Mouse Model

BALB/c nude mice (female, 6 weeks old) were used to evaluate the effects of AURKA PROTACs in vivo. Wild‐type KG1A cells were resuspended in PBS with a density of 5 × 10^6^/100 µL and subcutaneously inoculated into the right flank of the mice. After 6 days, dAurA383, dAurA450, PROTAC cocktail, and MLN8237 were intraperitoneally injected with an equal‐molar dosage of 30 µmol kg^−1^ day^−1^ (28.51 mg kg^−1^ day^−1^ for dAurA383, 29.16 mg kg^−1^ day^−1^ for dAurA450, and 15.57 mg kg^−1^ day^−1^ for MLN8237) in a 100 µL vehicle (DMSO 10%, PEG300 40%, Tween‐80 5%, saline 45%). The long axis (A) and short axis (B) of the tumor were measured every 2 days, the tumor volume *V* = *A* × *B* × *B*/2 was calculated, and the time–volume curve of tumor growth was plotted. After 2 weeks, blood was sampled from the mice's eyes for ALT, AST, and BUN testing. Then the mice were sacrificed and the tumors were dissected, weighted, and digested into single cells for flow cytometry and Western blot analysis.

### Cell Surface CD34 and CD11b Analysis

An equal number of cells were used for cell surface CD34 and/or CD11b staining together with the dead cell dye Zombie. In brief, DMSO, AURKA PROTACs, and ATRA (A9120, Solarbio) treated leukemia cells from the in vitro and in vivo assays were collected, counted, and washed with PBS. Subsequently, cells were resuspended and stained in a 100 µL staining buffer containing 1:100 diluted PE antihuman CD34 (343506, Biolegend) and/or 1:100 diluted Alexa Fluor 700 antimouse/human CD11b (101222, Biolegend) together with 1:500 diluted Zombie in the dark for 30 min at room temperature. After being washed with PBS, the cells were subjected to flow cytometry, while the relative cell surface CD34 and CD11b expression (MFI) were analyzed with CytExpert and FlowJo software.

### RNA Sequencing and Analysis

The AURKA PROTACs and DMSO treated cells were used for total RNA extraction with the HiPure Total RNA Plus Mini Kit and sent to the Novogene company. All samples prepared for the RNA‐SEQ library had RIN values greater than 9.5. An Illumina HiSeq2000 (150 bp, paired‐end) was used for sequencing. RNA‐seq data were analyzed using the RNACocktail toolkit.^[^ ^65^ ^]^ GSEA was executed with the GSEA algorithm.^[^ ^66^ ^]^

### Mass Spectrum and Analysis

The AURKA PROTACs and DMSO treated cells were collected, lysed, and quantified. An equal amount of peptide from each treatment was used for TMT labeling as DMSO‐TMT‐126, dAurA383‐TMT‐127, dAurA425‐TMT‐128, dAurA450‐TMT‐129, and cocktail‐TMT‐131. Labeled samples were mixed together and subjected to mass spectrum identification. Data were analyzed with the MaxQuant software and protein abundance was compared in the AURKA PROTACs treated versus DMSO treated cells. The top 100 decreased proteins were subjected to g:Profiler^[^ ^44^ ^]^ to execute GO analysis.

### ALDEFLUOR Assays

ALDH positive leukemia cells were analyzed with the ALDEFLUOR Stem Cell Identification Kit (01700, Stemcell) according to the manufacturer's protocol. In brief, leukemia cells were pretreated with the indicated concentration of AURKA PROTACs or DMSO for 2 days. Subsequently, cells were collected, washed, supplied with a 5 µL activated ALDEFLUOR substrate reagent and divided into two tubes as either the experimental group or the inactivated control group. The inactivated control group was further supplied with 5 µL ALDEFLUOR DEAB. All samples were incubated at 37 °C for 40 min in dark, then centrifuged and resuspended in 500 µL ALDEFLUOR buffer and subjected to flow cytometry. The proportion of ALDH positive cells was analyzed with DEAB treated group as control. Data were analyzed with CytExpert software.

### Study Approval

All animal studies and human subject research were approved by the ethical committee of Sun Yat‐sen University Cancer Center (IRB Approval Nos. L102012017220R and GZR2017‐041).

### Statistical Analysis and Data Availability

Statistical analyses were performed using GraphPad Prism 8 (GraphPad Software, Inc., LaJolla, CA, USA). Unpaired Student's *t*‐test was used to perform statistical analysis between the two groups. One‐Way ANOVA was used for comparisons between the two groups in multiple groups. The level of significance was set at a *P < 0.05, **P < 0.01, and ***P < 0.001. The data generated during and/or analyzed in this current study are available from the corresponding author upon reasonable request. The raw data for RNA‐Seq are available in the Genome Sequence Archive (Genomics, Proteomics & Bioinformatics 2017) database with the accession number: HRA001384. For manuscript reviewing, the shared URL is https://ngdc.cncb.ac.cn/gsa‐human/s/7F76qzm9 (temporary).

## Conflict of Interest

The authors declare no conflict of interest.

## Author Contributions

F.L., X.W., J.L.D., Z.J.H., Z.M.W., and L.L.L. contributed equally to this work. Conceptualization: ZF.W., SJ.W., Q.L., and F.L.; Methodology, F.L., ZF.W., X.W., JL.D., ZJ.H., ZM.W., LL.L., HQ.L., D.H., YF.R., Y.W., XY.L., JX.Z., ZJ.Z., B.H., M.Y., HM.Y. and LH.Z.; Investigation, Q.L., ZF.W., SJ.W. and JS.Y.; Software, ZF.W. and F.L.; Data Analysis, ZF.W. and Q.L.; Writing‐Review & Editing, ZF.W., F.L., Q.L., and SJ.W.; Supervision, Q.L. and ZF.W; Funding Acquisition, L.Q., SJ.W., ZF.W and M.Y.

## Supporting information

Supporting Information

Supplemental Table 1

Supplemental Table 2

## Data Availability

The data that support the findings of this study are openly available in Genome Sequence Archive at https://ngdc.cncb.ac.cn/gsa‐human/s/7F76qzm9, reference number 1384.
