# Supplementary material for: A Temporal PROTAC Cocktail‐Mediated Sequential Degradation of AURKA Abrogates Acute Myeloid Leukemia Stem Cells
Source: Adv Sci (Weinh). 2022 Jun 2;9(22):2104823. doi: 10.1002/advs.202104823 (PMC9353462; doi:10.1002/advs.202104823)
Supplement: Supplementary file 1 — Supporting Information [file ADVS-9-2104823-s003.pdf]

ADVANCED SCIENCE
Open Access

Supporting Information

for *Adv. Sci.*, DOI 10.1002/advs.202104823

A Temporal PROTAC Cocktail-Mediated Sequential Degradation of AURKA Abrogates Acute Myeloid Leukemia Stem Cells

Fang Liu, Xuan Wang, Jianli Duan, Zhijie Hou, Zhouming Wu, Lingling Liu, Hanqi Lei, Dan Huang, Yifei Ren, Yue Wang, Xinyan Li, Junxiao Zhuo, Zijian Zhang, Bin He, Min Yan, Huiming Yuan, Lihua Zhang, Jinsong Yan, Shijun Wen, Zifeng Wang* and Quentin Liu**

Supplemental Information

Chemistry

Synthesis of dAurA379

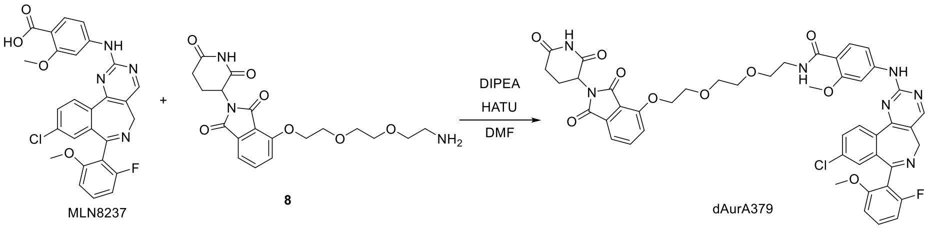

Compound **8** was prepared following the similar synthetic procedure for compound **2**. To a solution of compound **8** (35.0 mg) in DMF (1.0 mL) was added DIPEA (63 uL), MLN8237 (40.0 mg) and HATU (29.3 mg). The reaction mixture was stirred for 2h at room temperature. EtOAc was added, and the mixture washed with water three times. The organic layer was dried over $MgSO_4$, filtered and evaporated in Vacuo. The resulting residue was purified by silica column chromatography ($MeOH/CH_2Cl_2$ = 1/20) to provide dAurA379 as a yellow solid (40 mg, 56%). ^{1}H NMR (500 MHz, $CDCl_3$) δ 10.39 (s, 1H), 8.52 (s, 2H), 8.35 (s, 1H), 8.23 (d, *J* = 8.4 Hz, 1H), 8.11 (d, *J* = 8.6 Hz, 1H), 7.65 (t, *J* = 7.8 Hz, 1H), 7.58 (d, *J* = 8.4 Hz, 1H), 7.53 (s, 1H), 7.43 (m, 2H), 7.32 (s, 1H), 7.29 (d, *J* = 7.7 Hz, 1H), 7.24 (s, 1H), 6.75 (m, 2H), 4.99 (dd, *J* = 12.5, 5.0 Hz, 1H), 4.87 (brs, 1H), 4.34 (s, 2H), 3.99 - 3.87 (m, 4H), 3.84 (s, 3H), 3.78 (s, 2H), 3.70-3.60 (m, 6H), 3.34 (s, 1H), 2.94-2.82 (m, 2H), 2.75 (m, 1H), 2.13 (m, 1H). 1.99 (brs, 2H). LCMS (m/z, ESI): 905.3 (M + H^+). HPLC purity: 96.38%.

Synthesis of dAurA380

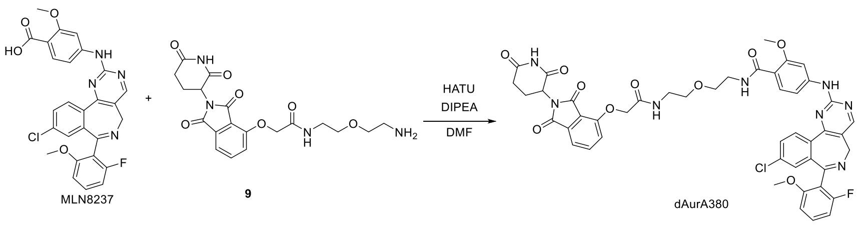

Compound **9** was prepared following the similar synthetic procedure for compound **2**. To a solution of compound **9** (38.7 mg) in DMF (1.0 mL) was added DIPEA (63 uL),

MLN8237 (40.0 mg) and HATU (29.3 mg). The reaction mixture was stirred for 2h at room temperature. EtOAc was added, and the mixture washed with water three times. The organic layer was dried over $MgSO_4$, filtered and evaporated in Vacuo. The resulting residue was purified by silica column chromatography ($MeOH/CH_2Cl_2$ = 1/20) to provide dAurA380 as a yellow solid (33.0 mg, 45%). ^1H NMR (500 MHz, $CDCl_3$) δ 10.21 (s, 1H), 8.44 (s, 1H), 8.40 (s, 1H), 8.23 (d, *J* = 8.4 Hz, 2H), 8.12 (d, *J* = 8.5 Hz, 1H), 7.89 (s, 1H), 7.68 (t, *J* = 7.8 Hz, 1H), 7.56 (d, *J* = 8.4 Hz, 1H), 7.49 (d, *J* = 7.3 Hz, 1H), 7.28 (m, 2H), 7.16 (d, *J* = 8.5 Hz, 1H), 7.12 (s, 1H), 6.67 (m, 2H), 4.88 (dd, *J* = 11.8, 5.1 Hz, 1H), 4.72 - 4.58 (m, 2H), 3.92 - 3.33 (m, 15H), 2.82 - 2.61 (m, 3H), 2.07 (m, 1H). LCMS (*m/z*, ESI): 918.2 (M + H^+). HPLC purity: 96.38%.

Synthesis of dAurA393

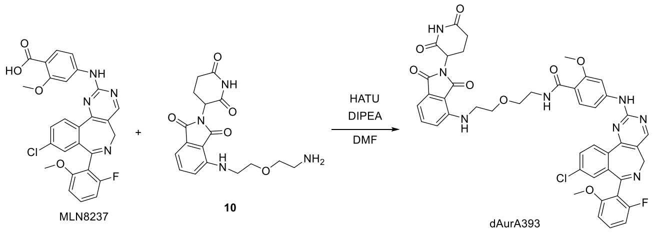

Compound **10** was prepared following the similar synthetic procedure for compoun **2**. To a solution of compound **10** (23.0 mg) in DMF (1.0 mL) was added DIPEA (52 uL), MLN8237 (33.0 mg) and HATU (24.2 mg). The reaction mixture was stirred for 2h at room temperature. EtOAc was added, and the mixture washed with water three times. The organic layer was dried over $MgSO_4$, filtered and evaporated in Vacuo. The resulting residue was purified by silica column chromatography ($MeOH/CH_2Cl_2$ = 1/20) to provide dAurA393 as a yellow solid (21 mg, 38%). ^1H NMR (400 MHz, $CDCl_3$) δ 9.60 (brs, 1H), 8.49 (s, 1H), 8.26 - 8.08 (m, 4H), 7.82 (s, 1H), 7.55 (dd, *J* = 8.5, 2.0 Hz, 1H), 7.50 - 7.42 (m, 1H), 7.30 (m, 2H), 7.20 (d, *J* = 8.4 Hz, 1H), 7.07 (d, *J* = 7.1 Hz, 1H), 6.91 (d, *J* = 8.6 Hz, 1H), 6.70 (brs, 2H), 6.55 (t, *J* = 5.2 Hz, 1H), 4.81 (s, 1H), 3.90 (m, 1H), 3.86 (s, 3H), 3.75 - 3.69 (m, 6H), 3.48 (m, 2H), 3.30 (brs, 1H), 2.84-2.62

(m, 3H), 2.10 - 1.94 (m, 3H). LCMS (*m/z*, ESI): 861.2 (M + H^+). HPLC purity: 97.46%.

Synthesis of dAurA408

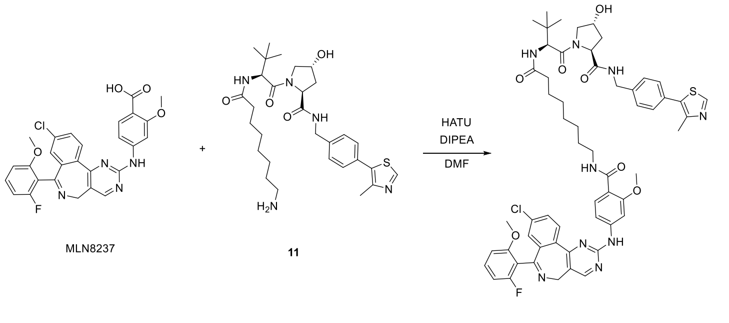

Compound **11** was prepared following the similar synthetic procedure for WZM421. To a solution of free amine **11** (27.6 mg) in DMF (1.0 mL) was added DIPEA (40.0 uL), MLN8237 (25.0 mg) and HATU (18.3 mg). The reaction solution was stirred for 2h at room temperature. EtOAc was added, and the mixture was washed with water three times. The organic layer was dried over $MgSO_4$, filtered and evaporated in Vacuo. The resulting residue was purified by column chromatography ($MeOH/CH_2Cl_2$ = 1/20) to provide WZM408 as a yellow solid (30.0 mg, 58%). ^1H NMR (500 MHz, *$CDCl_3$*) δ 8.83 (s, 1H), 8.60 (s, 1H), 8.43 (s, 1H), 8.14 (d, *J* = 8.4 Hz, 1H), 8.01 (d, *J* = 8.5 Hz, 1H), 7.93 - 7.79 (m, 3H), 7.49 (m, 2H), 7.22 (m, 4H), 7.04 (d, *J* = 8.4 Hz, 1H), 6.62 (d, *J* = 71.4 Hz, 2H), 6.33 (d, *J* = 8.6 Hz, 1H), 4.65 (t, *J* = 7.9 Hz, 1H), 4.47 (m, 3H), 4.28 (dd, *J* = 15.1, 5.2 Hz, 1H), 3.99 (d, *J* = 11.1 Hz, 1H), 3.89 (s, 3H), 3.59 - 3.56 (m, 2H), 3.34 (m, 2H), 3.08 (m, 3H), 2.41 (s, 3H), 2.37 - 2.29 (m, 1H), 2.16 - 2.04 (m, 3H), 1.50 (m, 2H), 1.29 - 1.18 (m, 8H), 0.88 (s, 9H). LCMS (m/z, ESI): 1072.4 (M + H^+). HPLC purity: 99.17%.

Synthesis of dAurA448

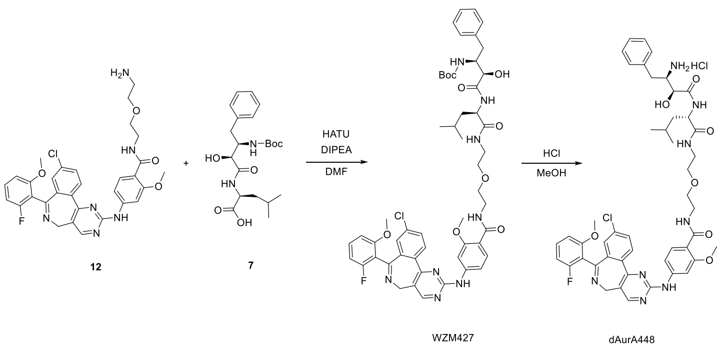

Compound **12** was prepared following the similar synthetic procedure for WZM444. To a solution of free amine **12** (55.0 mg) in DMF (1.0 mL) was added DIPEA (40.2 uL), **7** (44.6 mg) and HATU (34.6 mg). The reaction was stirred for 2h at room temperature. EtOAc was added, and the mixture was washed with water three times. The organic layer was dried over $MgSO_4$, filtered and evaporated in Vacuo.The resulting residue was purified by column chromatography ($MeOH/CH_2Cl_2$ = 1/20) to provide WZM427 as a yellow solid (36.0 mg, 40%). ^1H NMR (400 MHz, $CDCl_3$) δ 8.50 (s, 1H), 8.22 (d, *J* = 8.5 Hz, 1H), 8.12 (t, *J* = 5.9 Hz, 1H), 8.06 (d, *J* = 8.6 Hz, 1H), 7.98 (t, *J* = 12.5 Hz, 1H), 7.74 (s, 1H), 7.56 (dd, *J* = 8.5, 2.1 Hz, 1H), 7.34 - 7.27 (m, 2H), 7.22 (m, 4H), 7.13-7.00 (m, 2H), 6.68 (brs, 2H), 5.70 (s, 1H), 5.10 (t, *J* = 8.7 Hz, 1H), 4.87 (s, 1H), 4.52 (s, 1H), 4.28-4.10 (m, 2H), 3.99 (s, 3H), 3.74 (m, 1H), 3.67-3.40 (m, 6H), 3.28 (m, 1H), 2.96 (m, 1H), 1.90 (m, 2H), 1.75 - 1.46 (m, 4H), 1.34 (s, 9H), 0.87 (d, *J* = 6.3 Hz, 6H). LCMS (*m/z*, ESI): 995.4 (M + H^+). HPLC purity: 99.99%.

WZM427 (6.6 mg) was added to HCl solution in MeOH (1M, 1 mL). The reaction solution was stirred for 5h at room temperature. The mixture was concentrated in Vacuo to provide dAurA448 as a yellow solid (6.20 mg, 100%). LCMS (*m/z*, ESI): 931.2 (M + H^+). HPLC purity: 95.23%.

Synthesis of dAurA449

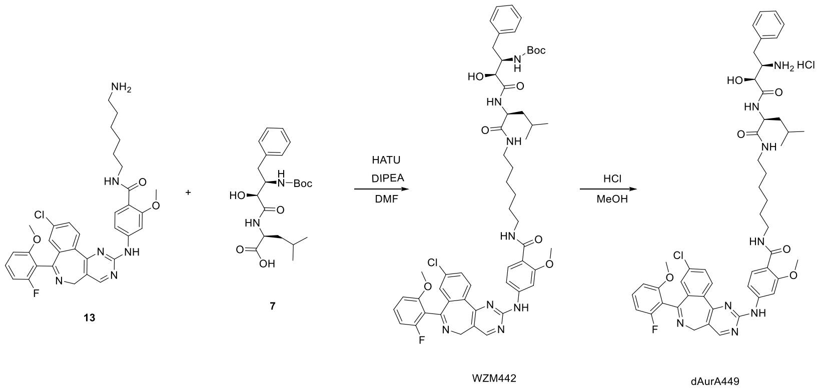

Compound **13** was prepared following the similar synthetic procedure for WZM444. To a solution of free amine **13** (30.0 mg) in DMF (1.0 mL) was added DIPEA (21.5 uL), **7** (23.8 mg) and HATU (18.5 mg). The reaction was stirred for 2h at room temperature. EtOAc was added, and the mixture was washed with water three times. The organic layer was dried over $MgSO_4$, filtered and evaporated in Vacuo. The resulting residue was purified by column chromatography ($MeOH/CH_2Cl_2$ = 1/20) to provide WZM442 as a yellow solid (34.0 mg, 69%). ^{1}H NMR (400 MHz, $CDCl_3$) δ 8.49 (s, 1H), 8.22 (d, *J* = 8.5 Hz, 1H), 8.06 (d, *J* = 8.5 Hz, 1H), 8.02 - 7.81 (m, 3H), 7.56 (dd, *J* = 8.5, 1.9 Hz, 1H), 7.32 (d, *J* = 1.7 Hz, 1H), 7.29 (d, *J* = 6.7 Hz, 1H), 7.18 (m, 6H), 7.03 - 6.88 (m, 1H), 6.63 (brs, 2H), 5.87 (s, 1H), 5.37 (s, 1H), 5.12 (d, *J* = 9.1 Hz, 1H), 4.86 (s, 1H), 4.59 - 4.39 (m, 1H), 4.28-4.09 (m, 3H), 3.97 (s, 3H), 3.50-3.25 (m, 5H), 3.05 - 2.83 (m, 3H), 1.78 - 1.11 (m, 13H), 0.89 (d, *J* = 6.4 Hz, 3H), 0.87 (d, *J* = 6.4 Hz, 3H). LCMS (m/z, ESI): 1007.4 ($M + H^+$). HPLC purity: 99.99%.

WZM442 (6.10 mg) was added to HCl solution in MeOH (1M, 1 mL). The reaction solution was stirred for 5h at room temperature. The mixture was concentrated in Vacuo to provide dAurA449 as a yellow solid (5.80 mg, 100%). LCMS (*m/z*, ESI): 907.4 ($M + H^+$). HPLC purity: 96.40%

Synthesis of dAurA1067

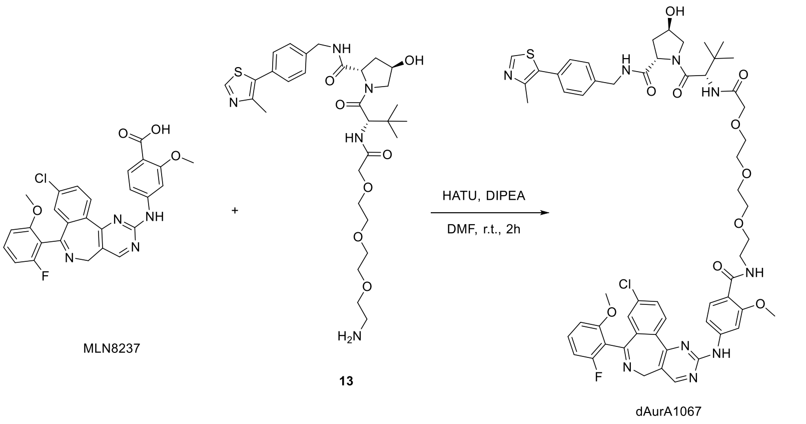

To a solution of free amine **13** (120.0 mg) in DMF (3.0 mL) was added DIPEA (90.0 uL), MLN8237 (100.5 mg) and HATU (88.3 mg). The reaction was stirred for 2h at room temperature. EtOAc was added, and the mixture was washed with water three times. The organic layer was dried over $MgSO_4$, filtered and evaporated in Vacuo. The resulting residue was purified by column chromatography ($MeOH/CH_2Cl_2$ = 1/20) to provide dAurA1067 as a yellow solid (98.0 mg, 45%). ^1H NMR (400 MHz, $CDCl_3$) δ 8.65 (s, 1H), 8.49 (s, 1H), 8.20 (d, *J* = 8.4 Hz, 2H), 8.08 (d, *J* = 8.6 Hz, 1H), 7.89 (d, *J* = 1.7 Hz, 1H), 7.77 (s, 1H), 7.65 - 7.58 (m, 1H), 7.55 (dd, *J* = 8.5, 2.1 Hz, 1H), 7.38 - 7.27 (m, 6H), 7.12 (d, *J* = 8.4 Hz, 1H), 6.68 (brs, 2H), 4.71 (t, *J* = 8.1 Hz, 1H), 4.62 - 4.47 (m, 3H), 4.31 (dd, *J* = 15.1, 5.4 Hz, 1H), 4.10 -3.94 (m, 3H), 3.93 (s, 3H), 3.75-3.55 (m, 13H), 2.48 (m, 2H), 2.40 (m, 1H), 2.16 (m, 1H), 1.90 (m, 2H), 0.97 (s, 9H). HPLC purity: 96.00%.

Synthesis of dAurA1071

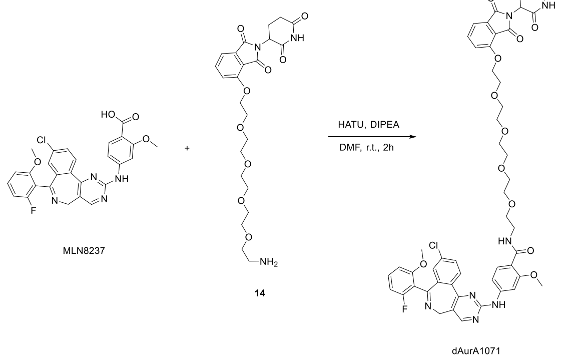

Compound **14** was prepared following the similar synthetic procedure for compound **2**. To a solution of free amine **14** (44.5 mg) in DMF (2.0 mL) was added DIPEA (42.0 uL), MLN8237 (42.0 mg) and HATU (41.0 mg). The reaction was stirred for 2h at room temperature. EtOAc was added, and the mixture was washed with water three times. The organic layer was dried over $MgSO_4$, filtered and evaporated in Vacuo. The resulting residue was purified by column chromatography ($MeOH/CH_2Cl_2$ = 1/20)to provide dAurA1071 as a yellow solid (33.0 mg, 37%). ^{1}H NMR (400 MHz, CDCl3) δ 9.87 (brs, 1H), 8.54 (s, 1H), 8.31 - 8.07 (m, 4H), 7.69 - 7.52 (m, 3H), 7.49 - 7.27 (m, 5H), 6.74 (m, 2H), 4.97 (dd, *J* = 12.1, 5.3 Hz, 1H), 4.85 (m, 1H), 4.33 (m, 2H), 3.91 (t, *J* = 4.4Hz, 2H), 3.85 (s, 3H), 3.73 (m, 2H), 3.66-3.62 (m, 13H), 2.92 - 2.68 (m, 3H), 2.13 (m, 1H). LCMS (*m/z*, ESI): 994.3 (M + H+). HPLC purity: 97.85%.

Synthesis of dAurA1072

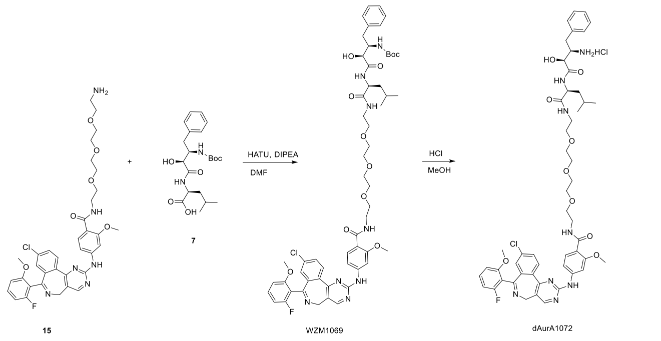

Compound **15** was prepared following the similar synthetic procedure for WZM444.To a solution of free amine **15** (52.0 mg) in DMF (2.0 mL) was added DIPEA (35.0 uL), **7** (27.6 mg) and HATU (31.4 mg). The reaction was stirred for 2h at room temperature. EtOAc was added, and the mixture was washed with water three times. The organic layer was dried over $MgSO_4$, filtered and evaporated in Vacuo. The resulting residue was purified by column chromatography ($MeOH/CH_2Cl_2$ = 1/20) to provide WZM1069 as a yellow solid (36.0 mg, 44%). WZM1069 (10.0 mg) was added to HCl solution in MeOH (1 M, 1 mL). The reaction solution was stirred for 5h at room temperature. The mixture was concentrated in Vacuo to provide dAurA1072 as a yellow solid (9.40 mg, 99%). LCMS (*m/z*, ESI): 983.4 (M + H^+). HPLC purity 97.38%.

Supplemental figure and table legend.

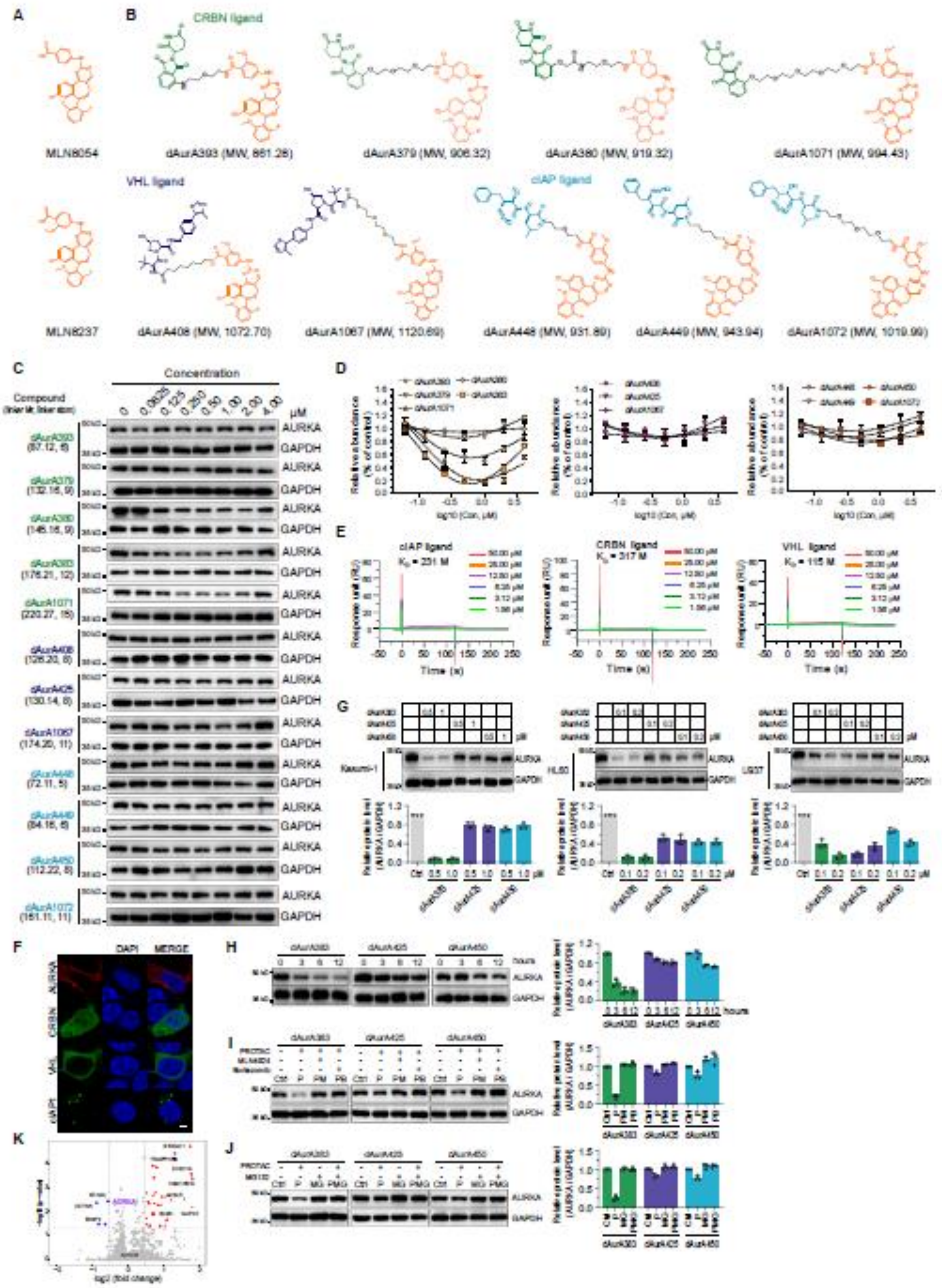

Figure S1. Design and characterization of different E3 ubiquitin ligase-based AURKA PROTACs.

A. Chemical structures of MLN8054 and MLN8237.

B. Chemical structures of AURKA PROTACs based on small molecule ligands for CRBN, VHL and cIAP. MW, molecular weight (Daltons).

C&D. Degradation of endogenous AURKA in KG1A cells treated with the indicated concentration of PROTACs for 6 hours. The relative amounts of each band were scanned and calculated relative to the lane's loading control. Linker Mr, relative molecular weight of linker (Daltons).

E. SPR sensorgrams of E3 ligands binding to AURKA protein.

F. HEK293T cells were transiently transfected with AURKA-mCherry-HOTag3, CRBN-EGFP-HOTag6, VHL-EGFP-HOTag6 or cIAP1-EGFP-HOTag6. Images were acquired with Zeiss LSM880 confocal microscope. Scale bar, 5 μm.

G. Degradation of endogenous AURKA in Kasumi-1, U937 and HL60 cells treated with indicated concentration of PROTACs for 6 hours. Relative AURKA protein levels were quantified using the Image J software.

H. Degradation of endogenous AURKA in KG1A cells treated with PROTACs (500 nM) for 0, 3, 6, or 12 hours. Relative AURKA protein levels were quantified using the Image J software.

I. Degradation of endogenous AURKA in KG1A cells treated with PROTACs (500 nM), NEDD8-activating enzyme inhibitor MLN4924 (1 μM) or proteasomal inhibitor Bortezomib (25 nM) for 12 hours. Relative AURKA protein levels were quantified using the Image J software.

J. Degradation of endogenous AURKA in KG1A cells treated with PROTACs (500 nM) or proteasomal inhibitor MG132 (6 μM) for 6 hours. Relative AURKA protein levels were quantified using the Image J software.

K. TMT-based quantitative proteomic after treatment with dAurA425 (500 nM) or DMSO for 6 hours in KG1A cells. The differential expressed proteins were presented in the volcano plot.

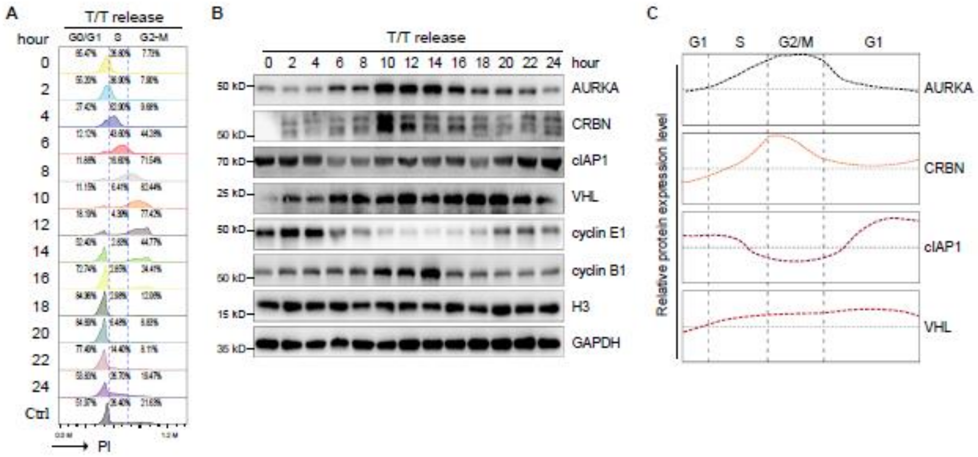

Figure S2. Characterization of E3 ubiquitin ligases in cell cycle.

A. Kasumi-1 cells were synchronized at the G1/S boundary by a double thymidine method, released into fresh media, and harvested at the indicated times (T/T release). The cell cycle profile was assayed by FACS with propidium iodide (PI) staining. Ctrl, proliferating Kasumi-1 cells under normal cell culture condition were used as control.

B. The protein expression levels of the indicated proteins after T/T release were measured by Western blot.

C. Schematic depiction of the relative protein levels of AURKA, CRBN, cIAP1, and VHL in the cell cycle.

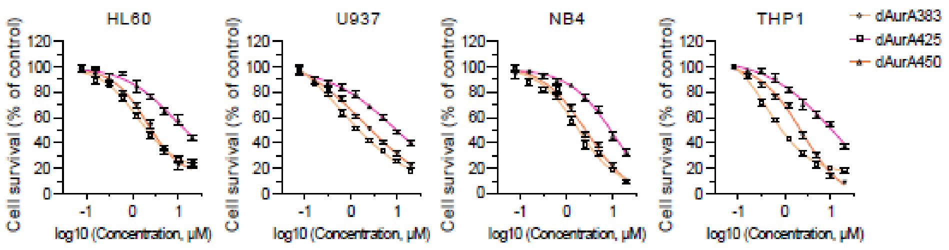

Figure S3. AURKA PROTACs inhibit cell growth of AML cells *in vitro*

Dose-response curves of HL60, U937, NB4 and THP1 treated with AURKA PROTACs. Cells were treated with various concentrations of AURKA PROTACs for 72 hours and were stained with CCK8. For IC50 in HL60 cells, dAurA383 = 1.33 ± 0.31 μM, dAurA425 = 7.28 ± 4.50 μM, dAurA450 = 2.01 ± 0.46 μM. For IC50 in

U937 cells, dAurA383 = 0.65 ± 4.35 μM, dAurA425 = 9.85 ± 7.03 μM, dAurA450 = 1.95 ± 0.92 μM. For IC50 in NB4 cells, dAurA383 = 1.70 ± 0.47 μM, dAurA425 = 11.50 ± 6.01 μM, dAurA450 = 2.77 ± 0.86 μM. For IC50 in THP1 cells, dAurA383 = 0.42 ± 0.17 μM, dAurA425 = 11.99 ± 9.07 μM, dAurA450 = 2.38 ± 0.49 μM.

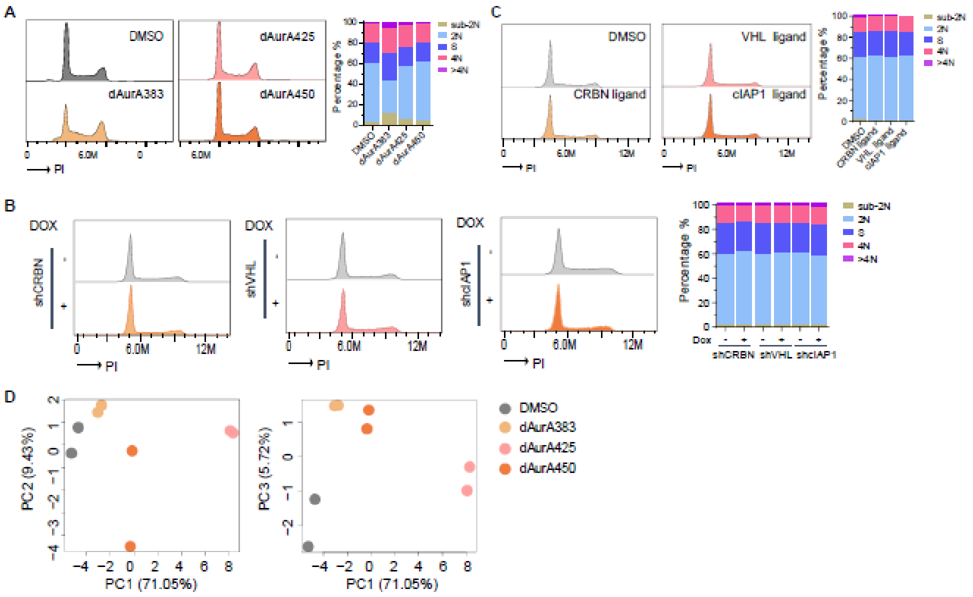

Figure S4. Characterization of cellular response to PROTACs in AML cells

A. Kasumi-1 cells were treated with AURKA PROTACs (1 μM) for 48 hours. Cell cycle profile was assayed by FACS with propidium iodide (PI) staining. The cell cycle distribution was analyzed by FlowJo software.

B. Knockdown of CRBN, VHL and cIAP1 with doxycycline (DOX, 0.5 μg/ml) for 48 hours in KG1A cells harboring inducible shRNA. Cell cycle profile was assayed by FACS with PI staining. The cell cycle distribution was analyzed by FlowJo software.

C. KG1A cells were treated with E3 ligase ligand (1 μM) for 48 hours. Cell cycle profile was assayed by FACS with PI staining. The cell cycle distribution was analyzed by FlowJo software.

D. The quality control of the RNA-seq data. Principal Component Analysis (PCA) plot was drawn to monitor the distribution and correlation of each sample.

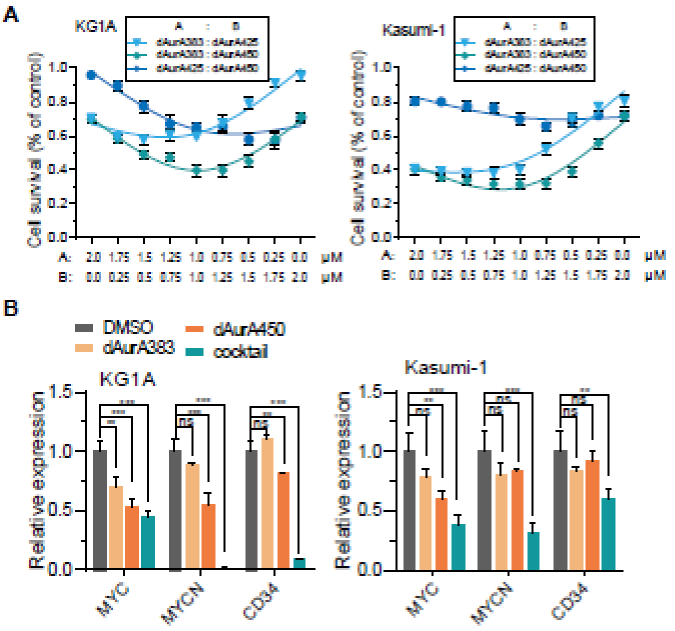

Figure S5. A dAurA383 and dAurA450 cocktail synergistically inhibit the growth and stemness of AML cells

A. The cells were treated with various ratios of PROTAC in combination for 72 hours. The growth inhibitory effects were determined by CCK8 staining.

B. Relative mRNA expression of MYC, NMYC and CD34 in DMSO, dAurA383, dAurA450 and PROTAC cocktail (1 μM) treated KG1A and Kasumi-1 cells.

Statistics, significance: one-way ANOVA with Bonferroni correction (C); ns, not significant; ***P* < 0.01; ****P* < 0.001.

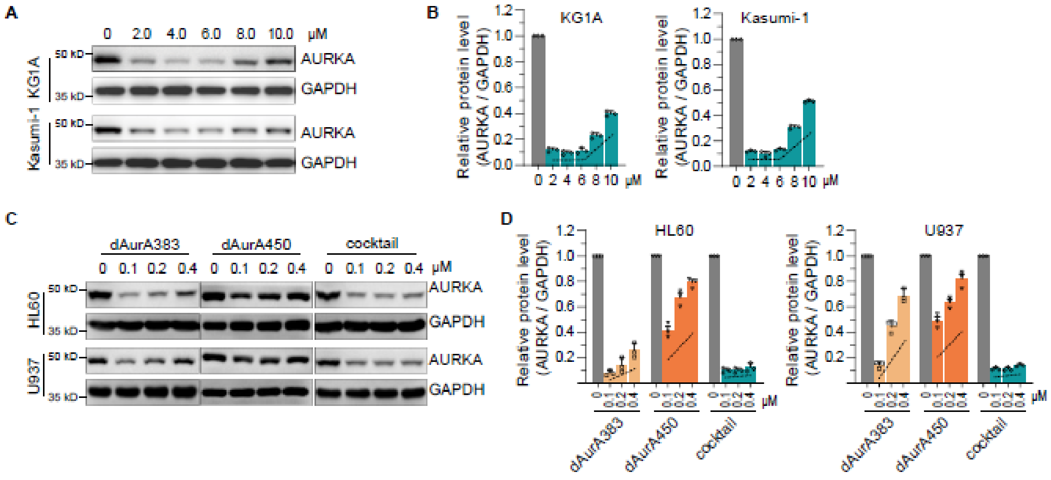

Figure S6. An AURKA PROTACs cocktail relieves the hook effect

A. Degradation of endogenous AURKA in KG1A and Kasumi-1 cells following 12 hours treatment with indicated concentration of PROTAC cocktail.

B. Relative AURKA protein levels in panel A were semi-quantitated using the Image J software.

C. Degradation of endogenous AURKA in HL60 and U937 cells following 12 hours treatment with indicated concentration of PROTACs.

D. Relative AURKA protein levels in panel C were semi-quantitated.

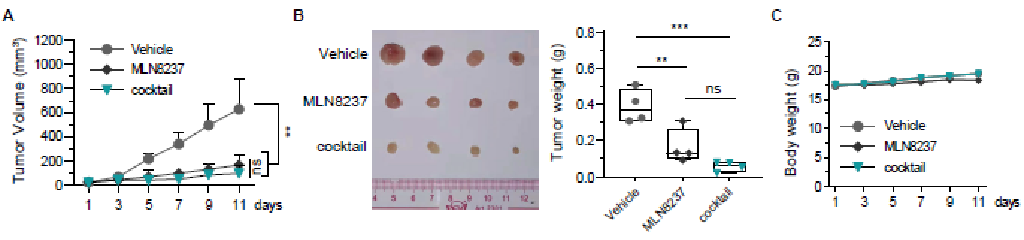

Figure S7. AURKA PROTAC cocktail induces AML regression in a xenograft mouse model

A. Nude mice bearing KG1A xenografts were intraperitoneally injected with PROTAC cocktail and MLN8237 (30 μmol/kg/day) for 11 days. The tumor volume from 1 to 11 days is plotted versus time.

B. Left panel, tumors resected from the mice in each group are shown. Right panel, statistical analysis of the tumor weight.

C. The body weight of the mice were measured and plotted against time.

Statistics, significance: one-way ANOVA with Bonferroni correction (A and B; ns, not significant; $**P < 0.01$; $***P < 0.001$.

Table S1. Normalized abundance of proteins in TMT-based quantitative proteomic assays

Table S2. Transcripts Per Million (TPM) of each gene

Table S3. Oligonucleotides of shRNA targeting CRBN, VHL and cIAP1

Table S4. Primers of qPCR assays

Table S3. Oligonucleotides of shRNA targeting CRBN, VHL and cIAP1

oligo	sequence (5'-3')
shCRBN-F	CCGGGGAAAGGGAAGCACAGTTTCTCGAGAAACTGTGCTTCCCTTTCCTTTTT
shCRBN-R	AATTAAAAAGGAAAGGGAAGCACAGTTTCTCGAG AAACTGTGCTTCCCTTTCC
shVHL-F	CCGGTATCACACTGCCAGTGTATACCTCGAGGTATACACTGGCAGTGTGATATTTTT
shVHL-R	AATTAAAAATATCACACTGCCAGTGTATACCTCGAGGTATACACTGGCAGTGTGATA
shcIAP1-F	CCGGGCCGAATTGTCTTTGGTGCTTCTCGAGAAGCACCAAAGACAATTCGGCTTTTT
shcIAP1-R	AATTAAAAAGCCGAATTGTCTTTGGTGCTTCTCGAGAAGCACCAAAGACAATTCGGC

Table S4. Primers of qPCR assays

primer	sequence
qCMYC-F	CCTGGTGCTCCATGAGGAGAC
qCMYC-R	CAGACTCTGACCTTTTGCCAGG
qNMYC-F	ACCACAAGGCCCTCAGTACCTC
qNMYC-R	TGACAGCCTTGGTGTTGGAGGA
qCD34-F	CCTCAGTGTCTACTGCTGGTCT
qCD34-R	GGAATAGCTCTGGTGGCTTGCA
qGAPDH-F	GTCTCCTCTGACTTCAACAGCG
qGAPDH-R	ACCACCCTGTTGCTGTAGCCAA
